# Sexually dimorphic activation of innate antitumor immunity prevents adrenocortical carcinoma development

**DOI:** 10.1126/sciadv.add0422

**Published:** 2022-10-14

**Authors:** James J. Wilmouth, Julie Olabe, Diana Garcia-Garcia, Cécily Lucas, Rachel Guiton, Florence Roucher-Boulez, Damien Dufour, Christelle Damon-Soubeyrand, Isabelle Sahut-Barnola, Jean-Christophe Pointud, Yoan Renaud, Adrien Levasseur, Igor Tauveron, Anne-Marie Lefrançois-Martinez, Antoine Martinez, Pierre Val

**Affiliations:** ^1^ Institut GReD (Genetics, Reproduction and Development), CNRS UMR 6293, Inserm U1103, Université Clermont Auvergne, 28 Place Henri Dunant, 63000 Clermont-Ferrand, France.; ^2^ Laboratoire de Biochimie et Biologie Moléculaire, UM Pathologies Endocriniennes, Groupement Hospitalier Est, Hospices Civils de Lyon, Bron, France.; ^3^ Université Claude Bernard Lyon 1, Lyon, France.; ^4^ Endocrinologie Diabétologie CHU Clermont Ferrand, 58 rue Montalembert, F63000 Clermont-Ferrand, France.

## Abstract

Unlike most cancers, adrenocortical carcinomas (ACCs) are more frequent in women than in men, but the underlying mechanisms of this sexual dimorphism remain elusive. Here, we show that inactivation of *Znrf3* in the mouse adrenal cortex, recapitulating the most frequent alteration in ACC patients, is associated with sexually dimorphic tumor progression. Although female knockouts develop metastatic carcinomas at 18 months, adrenal hyperplasia regresses in male knockouts. This male-specific phenotype is associated with androgen-dependent induction of senescence, recruitment, and differentiation of highly phagocytic macrophages that clear out senescent cells. In contrast, in females, macrophage recruitment is delayed and dampened, which allows for aggressive tumor progression. Consistently, analysis of TCGA-ACC data shows that phagocytic macrophages are more prominent in men and are associated with better prognosis. Together, these data show that phagocytic macrophages are key players in the sexual dimorphism of ACC that could be previously unidentified allies in the fight against this devastating cancer.

## Teaser

Androgen-dependent senescence induces recruitment of phagocytic macrophages, preventing adrenocortical carcinoma development.

## INTRODUCTION

Apart from reproductive tissues, cancer incidence and mortality are higher in males than in females (*1*, *2*). Together with thyroid cancer (*3*), adrenocortical carcinoma (ACC), which arises from steroidogenic cells of the adrenal cortex, is one of the rare exceptions to this rule. ACC female-to-male ratios range from 1.5 to 2.5:1, and women are generally diagnosed at a younger age (fig. S1A) (*4*–*7*). Although the higher rate of steady-state proliferation and more efficient adrenal cortex renewal in females (*6*, *8*, *9*) may play a role in sexually dimorphic tumorigenesis, the mechanisms underlying female prevalence of ACC remain elusive.

ACC is an aggressive cancer, with about 35% of patients presenting with metastatic disease at diagnosis. Overall, 5-year survival rates range between 16 and 47% and decrease to around 10% for metastatic patients (*10*). In line with the steroidogenic activity of the adrenal cortex, ACC is associated with hormonal hypersecretion in more than 50% of patients (*11*). The vast majority of secreting ACC produce excess glucocorticoids, but some tumors also produce sex steroids or, in some rare instances, aldosterone (*12*).

Radical surgical resection of ACC is the most effective therapeutic strategy for localized tumors, but the risk of recurrence remains high (*12*). In patients with advanced inoperable or metastatic ACC, the adrenolytic compound mitotane, a derivate of the insecticide DDT (dichloro-diphenyl-trichloroethane), remains the standard of care, used as a single agent or in combination with an etoposide-doxorubicin-platin polychemotherapy, depending on prognostic factors (*13*–*16*). Although these treatments can improve recurrence-free survival, their benefit on overall survival is still debated (*12*, *13*, *17*–*19*). Several phase 1/2 clinical trials of immune checkpoint inhibitors targeting PD-1 and PD-L1 have also been conducted in ACC patients (*20*–*23*). Unfortunately, these were associated with low response rates and have failed to improve patient outcome substantially. One potential reason for these modest results is the low level of lymphocyte infiltration in ACC (*24*), which appears to be associated with local production of glucocorticoids (*25*).

Understanding the molecular underpinnings of ACC pathogenesis is thus of utmost importance to develop novel therapeutic approaches. Large-scale pan-genomic studies have identified homozygous deletion of *ZNRF3* as the most frequent genetic alteration in ACC (*26*, *27*). This gene encodes a membrane E3 ubiquitin ligase that inhibits WNT signaling by inducing ubiquitination and degradation of Frizzled receptors (*28*, *29*). We previously showed that conditional ablation of *Znrf3* within steroidogenic cells of the adrenal cortex resulted in moderate WNT pathway activation and adrenal zona fasciculata hyperplasia up to 6 weeks, suggesting that ZNRF3 was a potential tumor suppressor in the adrenal cortex (*30*). However, we did not evaluate later stages of tumor progression.

Here, we show that tumor progression following ablation of *Znrf3* within steroidogenic cells of the adrenal cortex is sexually dimorphic. Whereas most female mice develop full-fledged metastatic carcinomas over an 18-month time course, adrenal hyperplasia gradually regresses in male knockout (KO) mice. We show that male-specific regression of hyperplasia is associated with induction of senescence, recruitment of macrophages, and differentiation of active phagocytes that clear out senescent steroidogenic cells. Although some degree of macrophage recruitment is observed in female mice, it is delayed and dampened compared to males, which allows for tumor progression. This phenomenon is dependent on androgens and can be triggered by testosterone treatment in females. Although macrophages are present within adrenal tumors at 18 months, active phagocytes, characterized by expression of the TYRO-3, AXL and MER family (TAM) receptor MERTK, are mostly found in males but not in females. Consistent with our observations in mice, analysis of RNA sequencing data from The Cancer Genome Atlas (TCGA) cohort of ACC shows that phagocytic macrophages are more prominent in men than in women and are associated with better prognosis. Together, these data establish that phagocytic macrophages prevent aggressive ACC development in male mice and suggest that they may play a key role in the unusual sexual dimorphism of ACC in patients.

## RESULTS

### Tumor progression in *Znrf3 cKO* adrenals is sexually dimorphic

**Fig. 1.**
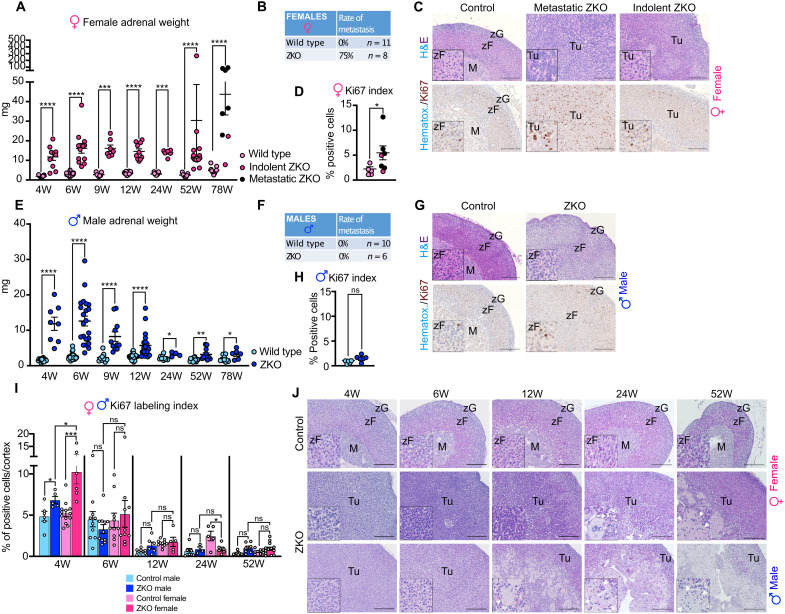
Sexually dimorphic tumor progression in *Znrf3 cKO* adrenals. (**A**) Female adrenal weights measured from 4 to 78 weeks in wild-type and *Znrf3 cKO* (ZKO) adrenals. (**B**) Rate of metastasis in 78-week-old *Znrf3 cKO* females. (**C**) Histology (top) and immunohistochemical (IHC) analysis of Ki67 expression (bottom) in 78-week-old female controls, *Znrf3 cKO* adrenals associated with metastasis formation, or indolent *Znrf3 cKO* adrenals. (**D**) Quantification of the Ki67 proliferation index as the ratio of positive cells over total nuclei in the cortex of 78-week-old control and *Znrf3 cKO* females. (**E**) Male adrenal weights measured from 4 to 78 weeks in wild-type and *Znrf3 cKO* (ZKO) adrenals. (**F**) Rate of metastasis in 78-week-old *Znrf3 cKO* males. (**G**) Histology (top) and IHC analysis of Ki67 expression (bottom) in 78-week-old male controls and *Znrf3 cKO* adrenals. (**H**) Quantification of the Ki67 proliferation index as the ratio of positive cells over total nuclei in the cortex of 78-week-old control and *Znrf3 cKO* males. (**I**) Kinetic analysis of the Ki67 proliferation index from 4 to 52 weeks in male and female control and *Znrf3 cKO* adrenals. (**J**) Kinetic analysis of the histological phenotype from 4 to 52 weeks in male and female control and *Znrf3 cKO* adrenals. Arrowheads in insets show multinucleated giant cells (MGCs) that accumulate in the inner cortex of mutant male mice and, to a lesser extent, mutant female mice. M, medulla; zF, zona fasciculata; zG, zona glomerulosa; Tu, tumor. Scale bars, 200 μm. Graphs represent means ± SEM. Statistical analyses were conducted by Mann-Whitney tests in (A), (D), (E), and (H) and by two-way ANOVA in (I). ns, not significant; *P < 0.05; **P < 0.01; ***P < 0.001; ****P < 0.0001.

We previously showed that adrenal targeted ablation of *Znrf3* resulted in strong zona fasciculata hyperplasia at 6 weeks of age, but we did not evaluate the phenotype at later stages (*30*). To gain further insight into the potential tumor suppressor function of ZNRF3 in the adrenal cortex, we conducted a kinetic analysis from 4 to 78 weeks (Fig. 1). In female *Znrf3 cKO* mice (ZKO) (fig. S1B), adrenal weight increased progressively from 4 to 6 weeks and remained higher from 9 to 52 weeks. At 78 weeks, a majority of female *Znrf3 cKO* adrenals showed a more than sevenfold increase in weight compared to controls (ZKO median, 35.3 mg versus 4.6 mg for control; Fig. 1A). This suggested malignant transformation of adrenals over time. Consistent with this idea, introduction of the mTmG reporter in the breeding scheme (fig. S1B) allowed identification of multiple micro- and macrometastases in the local lymph nodes, peritoneal cavity, liver, and lungs of 75% of female ZKO at 78 weeks (Fig. 1B and fig. S1C). Histological analysis of adrenals that were associated with metastatic development (Fig. 1C) showed complete disorganization of the cortex that was mostly composed of densely packed small basophilic cells. This was associated with a significant increase in Ki67 labeling index (Fig. 1, C and D), although proliferation was rather heterogeneous throughout the tumor with areas showing up to 25% Ki67 labeling (fig. S1D). In contrast, in the few mutant mice where no metastases were found at 78 weeks (indolent ZKO), adrenals were largely hyperplastic, but cells retained a relatively normal morphology and Ki67 labeling was similar to control (Fig. 1C). Together, these data suggested that ZNRF3 behaved as a classical tumor suppressor in female mice, its ablation resulting in a high frequency of aggressive ACC formation at 78 weeks. In sharp contrast, although male *Znrf3 cKO* adrenals were also larger at 4 and 6 weeks, adrenal weight steadily declined thereafter, almost returning to normal at 78 weeks (Fig. 1E). This was associated with lack of metastatic progression (Fig. 1F), benign histology, and low Ki67 labeling index (Fig. 1, G and H), although some patches of higher proliferation could be detected in some adrenals (fig. S1D). This suggested that overall tumor development was rapidly blunted in males, although the initial hyperplastic phase was equivalent to females.

To further gain insight into this sexually dimorphic phenotype, we evaluated proliferation from 4 to 52 weeks. Analysis of Ki67 labeling index showed that following an early significant increase, both males and females had a rapid arrest in proliferation from 6 weeks onward (Fig. 1I and fig. S1E). The steady decline in adrenal weight, despite comparable proliferation in male KO adrenals and controls after 4 weeks, suggested that an active mechanism counteracted tumor progression in males. Unexpectedly though, there was no increase in apoptosis, measured by cleaved caspase-3 staining, in either female or male adrenals at 6 and 12 weeks (fig. S1F). To try to further understand the sexually dimorphic phenotype, we conducted a careful kinetic evaluation of adrenal histology. This showed a similar hyperplastic phenotype in males and females at 4 and 6 weeks (Fig. 1J). Hyperplasia progressed in females with accumulation of small basophilic cells that composed most of the gland by 52 weeks (Fig. 1J). Notably, starting at 12 weeks, we observed progressive thinning of the cortex (eosinophilic cells) and concomitant appearance and expansion of multinucleated giant cells (MGCs; containing up to 12 nuclei per cell) that progressively took over a large proportion of the male *Znrf3 cKO* gland (up to 40%) (Fig. 1J). In females, some MGCs were also observed. However, they were first visible at 24 weeks and only represented a small proportion of the gland, even at 52 weeks (Fig. 1J). MGCs were reminiscent of fused macrophages that are observed in granulomatous inflammatory diseases, which suggested a potential involvement of innate immune cells in preventing tumor progression in male *Znrf3 cKO* adrenals.

### Regression in male *Znrf3 cKO* adrenals is correlated with macrophage infiltration and fusion

**Fig. 2.**
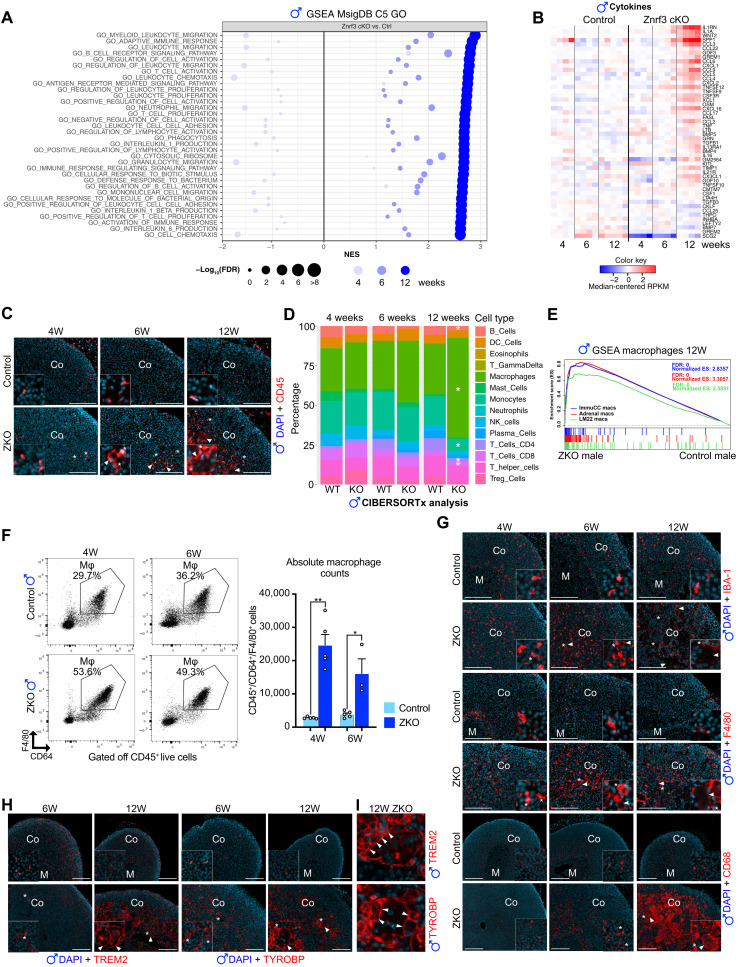
Regression in male *Znrf3 cKO* adrenals is correlated with macrophage infiltration and fusion. (**A**) GSEA of gene expression data from 4-, 6-, and 12-week-old control and *Znrf3 cKO* male adrenals. Plot represents the 35 gene sets from the C5 Gene Ontology database, with the highest enrichment score in *Znrf3 cKO* adrenals at 12 weeks. NES, normalized enrichment score. (**B**) Expression of cytokine/chemokine-coding genes in control and *Znrf3 cKO* adrenals at 4, 6, and 12 weeks. (**C**) IHC analysis of CD45 expression in adrenals from control and *Znrf3 cKO* (ZKO) mice at 4, 6, and 12 weeks. Stars show mononucleated leukocytes. (**D**) Stacked bar plots representing immune cell populations deconvoluted using CIBERSORTx and the LM22 expression matrix, from gene expression data in control and *Znrf3 cKO* male adrenals at 4, 6, and 12 weeks. WT, wild type. (**E**) GSEA of macrophage signatures derived from ImmuCC, LM22, and scRNA-seq of mouse adrenals (adrenal macs) (*51*) in 12-week-old male *Znrf3 cKO* adrenals. (**F**) Left: Dot plots of flow cytometry analysis of macrophage infiltration (defined as CD45^+^/CD64^+^/F4/80^+^ live cells) in 4- and 6-week-old control (top) and *Znrf3 cKO* (bottom) adrenals. Right: Quantification of absolute numbers of macrophages by flow cytometry. (**G**) IHC analysis of pan-macrophage markers IBA-1, F4/80, and CD68 in 4-, 6-, and 12-week-old control and *Znrf3 cKO* adrenals. (**H**) IHC for macrophage fusion–associated markers TREM2 and TYROBP in 6- and 12-week-old control and *Znrf3 cKO* adrenals. (**I**) High-magnification images of TREM2 and TYROBP staining showing fusion of mononucleated with multinucleated macrophages in *Znrf3 cKO* adrenals at 12 weeks. In (C) and (G) to (I), arrowheads show MGCs and stars show mononucleated macrophages. Co, cortex. Scale bars, 200 μm. Graphs represent means ± SEM. Statistical analyses in (F) were conducted by Mann-Whitney tests. *P < 0.05; **P < 0.01. DAPI, 4′,6-diamidino-2-phenylindole.

To further gain insight into the underpinnings of the regression phenomenon, we analyzed global gene expression by bulk RNA sequencing of control and *Znrf3 cKO* male adrenals at 4, 6, and 12 weeks. Gene set enrichment analysis (GSEA) of the RNA sequencing data using the C5 Gene Ontology (GO) database showed that at 12 weeks, the 34 most significantly enriched gene sets were all related with immune response and inflammation (Fig. 2A). Most of these gene sets were either not [false discovery rate (FDR) > 0.05] or negatively enriched at 4 weeks and showed an intermediate enrichment score at 6 weeks. This suggested that ablation of *Znrf3* resulted in the progressive establishment of a proinflammatory environment. Consistent with this idea, a large number of proinflammatory cytokines and chemokines genes were progressively up-regulated at 6 and 12 weeks (Fig. 2B and fig. S2A). Establishment of an inflammatory environment was further evaluated by immunohistochemistry (IHC) for the pan-leukocyte marker CD45. In control male adrenals, a few CD45^+^ cells were found scattered throughout the cortex. Four-week-old male *Znrf3 cKO* adrenals were similar to controls, although more mononucleated leukocytes were present in the inner cortex. At 12 weeks, the number of CD45-positive cells markedly increased in KO adrenals (Fig. 2C and fig. S2B). These comprised both mononuclear cells (stars) and the MGCs (arrowheads) that accumulated in the inner cortex (Fig. 2C). To further identify the immune cell types that composed the infiltrate, we deconvoluted RNA sequencing data with CIBERSORTx (*31*) using immune cell signatures from ImmuCC (Fig. 2D) (*32*) and mMCP (fig. S2C) (*33*). Both approaches showed a significant increase in macrophage populations, which represented 63% of all immune populations at 12 weeks. This was further confirmed by GSEA, showing a highly significant positive enrichment of multiple macrophage signatures at 12 weeks (Fig. 2E) and by reverse transcription quantitative polymerase chain reaction (RT-qPCR) showing a progressive accumulation of the macrophage marker transcripts *Cd68*, *Adgre1*, and *Cd11b* (fig. S2D). Together, these data strongly suggested that regression of adrenal cortex hyperplasia in *Znrf3 cKO* males was associated with establishment of a proinflammatory environment and abundant recruitment of macrophages.

To further confirm the nature of infiltrating cells, adrenals from control and *Znrf3 cKO* males were dissociated and analyzed by flow cytometry (Fig. 2F and fig. S2E). This showed that absolute numbers of CD45^+^/CD64^+^/F4/80^+^ macrophages were markedly increased in *Znrf3 cKO* adrenals at 4 and 6 weeks (Fig. 2F). Increased infiltration of macrophages in *Znrf3 cKO* male adrenals was further confirmed when macrophages were evaluated as a percentage of CD45^+^ cells (fig. S2F). Flow cytometry analyses further showed that at 4 weeks, almost 80% of CD45^+^/CD64^+^ macrophages coexpressed the M1 markers CD38 and major histocompatibility complex II (MHC-II), together with the M2 marker CD206, in both wild-type and *Znrf3 cKO* adrenals (fig. S3A). Although there was a very mild but significant increase in both MHC-II^+^/CD206^+^ and CD38^+^/CD206^+^ double-positive macrophages in 6-week *Znrf3 cKO* adrenals, there was no significant difference in either M1 or M2 macrophage proportions, following ablation of *Znrf3* at the two analyzed stages (fig. S3A). RT-qPCR (fig. S3B) and RNA sequencing analyses (fig. S3, C and D) further confirmed deregulation of both M1 and M2 markers in *Znrf3 cKO* adrenals, indicating that infiltrating macrophages had mixed M1 and M2 characteristics at 4 and 6 weeks.

Unfortunately, most of the CD45^+^ MGCs that accumulated from 12 weeks onward had a cell diameter larger than 40 μm, which precluded their characterization by flow cytometry (fig. S4A). To further characterize immune infiltration during the regression period, we thus resorted to IHC analysis. Staining with pan-macrophage markers IBA-1 and F4/80 confirmed progressive infiltration from 4 to 12 weeks (Fig. 2G and fig. S4B). Although mononuclear cells appeared equivalently labeled by both IBA-1 and F4/80, IBA-1 staining of MGCs was weak compared to F4/80 (Fig. 2G). However, MGCs displayed high levels of cytoplasmic CD68 staining, suggesting that they were derived from the fusion of mononuclear macrophages (Fig. 2G and fig. S4B). Macrophage fusion has been shown to rely on TREM2, an activating receptor of the immunoglobulin superfamily, and on TYROBP/DAP12, its transmembrane signaling adaptor (*34*, *35*). Expression of *Trem2* and *Tyrobp/Dap12* was strongly increased in RT-qPCR at 12 weeks (fig. S4C), and IHC analyses showed a strong up-regulation of both TREM2 and TYROBP protein accumulation in MGCs (Fig. 2H and fig. S4D). High-magnification images further showed TREM2/TYROBP-positive mononuclear macrophages actively fusing with MGCs (Fig. 2I, arrowheads). Together, this suggested that *Znrf3* ablation in adrenocortical cells resulted in macrophage infiltration and fusion to form MGCs in male adrenals.

### Infiltrating macrophages actively phagocytose steroidogenic cells

**Fig. 3.**
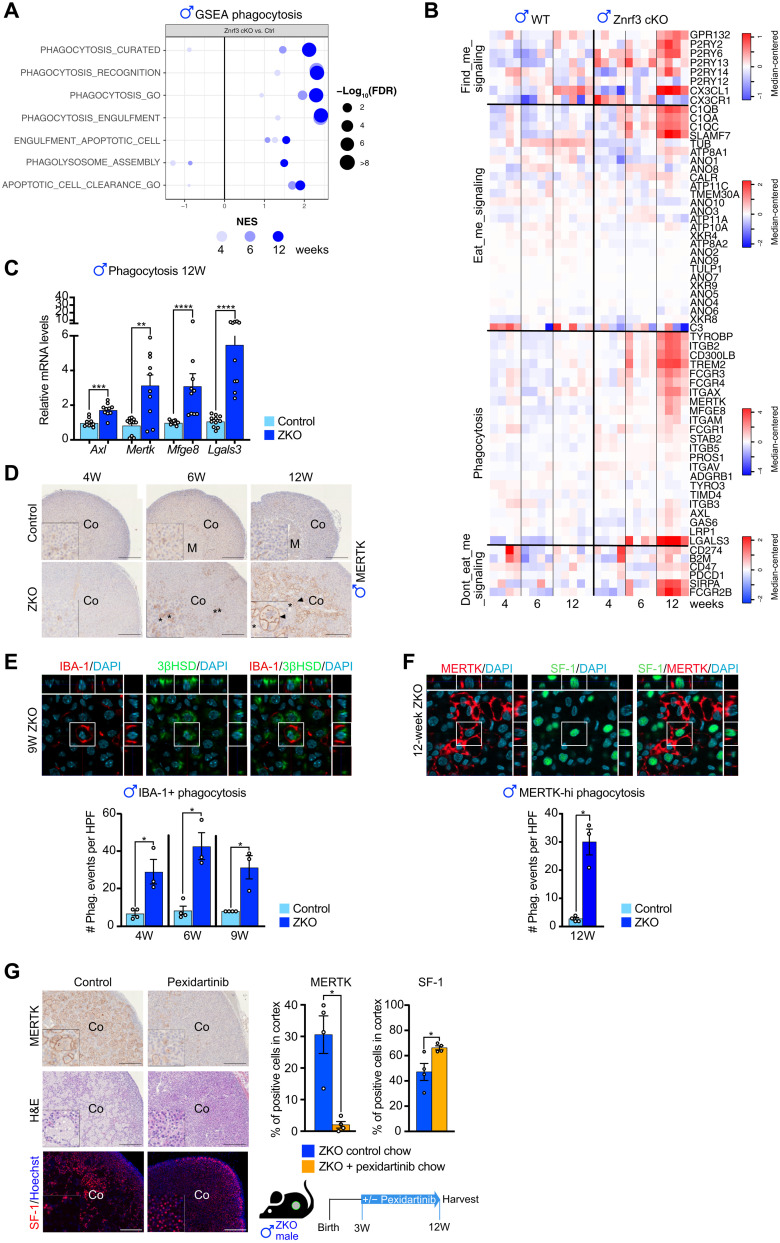
Infiltrating macrophages actively phagocytose steroidogenic cells. (**A**) GSEA of gene expression data from 4-, 6-, and 12-week-old control and *Znrf3 cKO* male adrenals. The plot represents enrichment of phagocytosis/efferocytosis gene sets in *Znrf3 cKO* male adrenals. (**B**) Expression of key regulators of the phagocytic pathway in control and *Znrf3 cKO* male adrenals at 4, 6, and 12 weeks. (**C**) RT-qPCR analysis of the expression of phagocytosis-associated genes in control and *Znrf3 cKO* male adrenals at 12 weeks. (**D**) IHC for the phagocytosis receptor MERTK in control and *Znrf3 cKO* male adrenals at 4, 6, and 12 weeks. Arrowheads show multinucleated macrophages, and stars show mononucleated macrophages. (**E** and **F**) Evaluation of phagocytosis by IHC for 3βHSD (steroidogenic cells) and IBA-1 (E) or SF-1 (steroidogenic cells) and MERTK (F). Images were acquired by confocal microscopy, and phagocytic events were counted when steroidogenic markers were found within the boundaries of macrophage markers along the z-stack. Panels show representative zoomed-in images (×120) in 9-week-old (IBA-1) and 12-week-old (MERTK) *Znrf3 cKO* adrenals. White boxes show phagocytic events on the two-dimensional projection of z-stack and within the orthogonal projections (side images). Bottom graphs represent quantification of phagocytic events on 10 high-power fields (HPFs; ×40) per individual mouse from 4 to 9 weeks (IBA-1^+^ phagocytosis) and at 12 weeks (MERTK^high^ phagocytosis). (**G**) IHC (MERTK and SF-1) and histological (H&E) analysis of *Znrf3 cKO* male mice that received control or pexidartinib-enriched chow from 3 to 12 weeks (left). Percentages of MERTK-positive and SF-1–positive cells, relative to total cortical cell numbers (DAPI^+^), are displayed on the graphs (right). Scale bar, 200 μm. Graphs represent means ± SEM. Statistical analyses in (C) and (E) to (G) were conducted by Mann-Whitney tests. *P < 0.05; **P< 0.01; ***P < 0.001; ****P < 0.0001.

Macrophages have been suggested to play a role in the early response to oncogenic insult by clearing out preneoplastic cells (*36*, *37*). GSEA of RNA sequencing data showed a progressive significant enrichment of gene sets associated with phagocytosis and clearance of apoptotic cells in male *Znrf3 cKO* adrenals, suggesting a potential role of phagocytosis in regression of hyperplasia (Fig. 3A). Phagocytosis involves chemotaxis of macrophages toward target cells that express “find-me” signals and recognition of target cells through “eat-me” signals that can be received directly by phagocytic receptors or indirectly after opsonization. Detailed analysis of RNA sequencing data showed significant up-regulation of genes coding the potential find-me chemokine CX3CL1 (*38*) and of the GPR132/G2A and P2RY2/P2RY6 metabotropic receptors that recognize lysophosphatidylcholine (GPR132) (*39*) and nucleotides (P2RY2/P2RY6) (*40*) released by target cells (Fig. 3B). Among potential eat-me signals, we found significant overexpression of C1Q complement components *C1qa*, *C1qb*, and *C1qc*, which have been shown to decorate the surface of apoptotic cells to target them for phagocytosis (*40*, *41*) (Fig. 3B), and of *Slamf7*, which is involved in phagocytosis of hematopoietic tumor cells (*42*). There was also up-regulation of the gene coding MFGE8, which opsonizes apoptotic cells and is recognized by the integrin receptors α_v_β_3_ and α_v_β_5_ at the membrane of macrophages (Fig. 3, B and C) (*43*, *44*). TREM2 and TYROBP, which we found overexpressed at both the mRNA and protein level (Figs. 2H and 3B and fig. S4C), can also be involved in the phagocytic process through recognition of lipids and ApoE-opsonized cells (*43*, *45*, *46*). Among the three TAM receptor tyrosine kinases, which play a central role in phagocytosis (TYRO3, MERTK, and AXL) (*40*, *47*), *Mertk* was expressed at high levels and showed the most significant up-regulation in *Znrf3 cKO* adrenals (Fig. 3, B and C). Although there was no up-regulation of *Gas6* and *Pros1*, the natural TAM receptor ligands (*40*), there was a strong overexpression of *Lgals3* (27-fold), which encodes Galectin-3, a phosphatidylserine-independent MERTK-specific opsonin (*43*, *48*) (Fig. 3B). This was further confirmed by RT-qPCR (Fig. 3C), suggesting that engagement of MERTK by Galectin-3 may trigger phagocytosis of *Znrf3 cKO* hyperplastic steroidogenic cells.

To further gain insight into a potential phagocytic process in *Znrf3 cKO* adrenals, we analyzed expression of the TAM receptor MERTK by IHC. Although some positive cells were found in wild-type adrenals, they were rather scarce and expressed low levels of MERTK (Fig. 3D). In contrast, increased numbers of mononuclear MERTK^high^ cells were found in *Znrf3 cKO* adrenals as early as 6 weeks (Fig. 3D). Most of these cells also stained for IBA-1, confirming their macrophage identity (fig. S4E). At 6 and 12 weeks, the number of mononuclear MERTK-high cells markedly increased in *Znrf3 cKO* (Fig. 3D and fig. S4E). Multinucleated fused macrophages expressed very high levels of MERTK (Fig. 3D), which was associated with reduced IBA-1 expression (fig. S4E). MERTK^high^ and, in particular, fused macrophages were also positive for TREM2 (fig. S4F). However, TREM2 was expressed in a larger number of macrophages, including mononucleated MERTK^−^ macrophages (fig. S4F). Together, this suggested that macrophage infiltration in *Znrf3 cKO* male adrenals was associated with differentiation into active phagocytes.

To test this hypothesis, we evaluated phagocytosis by confocal microscopy. For this, we colocalized expression of 3βHSD and SF-1, two markers of steroidogenic cells with IBA-1 (from 4 to 9 weeks) and MERTK (at 12 weeks). We then counted 3βHSD and SF-1–positive cells that were found within the boundaries of IBA-1^+^ or MERTK^high^ macrophages throughout the confocal z-stack (Fig. 3E). A few IBA-1^+^ macrophages contained 3βHSD-positive cells in control adrenals at 4, 6, and 9 weeks, indicating that phagocytosis of steroidogenic cells was taking place at homeostasis in the adrenal (Fig. 3E). The number of phagocytic IBA-1^+^ cells was markedly increased in *Znrf3 cKO* adrenals at these three time points (Fig. 3E), indicating that mononuclear IBA-1^+^ macrophages were actively involved in phagocytosis of *Znrf3 cKO* steroidogenic cells. Increased phagocytosis was also observed for MERTK^high^ macrophages at 12 weeks (Fig. 3F). Together, these data show that both IBA-1^+^ and MERTK^high^ macrophages are involved in a marked increase in phagocytosis of mutant steroidogenic cells in male *Znrf3 cKO* adrenals.

To further confirm the key role of macrophages in regression of adrenal hyperplasia, we depleted macrophages using a diet enriched with pexidartinib (290 mg/kg), a pharmacological inhibitor of CSF1R. This tyrosine kinase receptor plays a central role for survival of macrophages within their tissue niches through stimulation by CSF1 and/or interleukin-34 (IL-34). Consistent with the key function of CSF1R, flow cytometry analyses showed that 1 week of pexidartinib chow was sufficient to deplete almost all CD45^+^/CD64^+^/F4/80^+^ macrophages within the adrenal cortex of control male mice (fig. S4G). We then evaluated the impact of macrophage depletion in male *Znrf3 cKO* mice by feeding them with standard chow or pexidartinib chow from 3 to 12 weeks (Fig. 3G). This resulted in a very strong decrease in the number of IBA-1^+^, F4/80^+^, CD68^+^ (fig. S4, H and I), and MERTK^high^ macrophages (Fig. 3G) in IHC analyses, which was confirmed by RT-qPCR for *Adgre1*, *Cd68*, and *Cd11b* (fig. S4J). Consistent with these findings, although adrenal weight remained equivalent to control *Znrf3 cKO* males (fig. S4K), hematoxylin and eosin (H&E) staining showed a remarkable decrease in the number of fused macrophages and concomitant expansion of presumptive eosinophilic steroidogenic cells, following pexidartinib treatment (Fig. 3G). This was further confirmed by a significant increase in SF-1–positive cells in the cortices of pexidartinib-treated mice (Fig. 3G) and an inverse correlation between MERTK^high^ and SF-1–positive cells (fig. S4L). Together, these data show that *Znrf3* ablation induces sustained recruitment of IBA-1^+^ and MERTK^high^ macrophages, which results in phagocytic clearance of preneoplastic steroidogenic cells and regression of adrenal hyperplasia in male mice.

### Recruitment of phagocytic macrophages is delayed in females

**Fig. 4.**
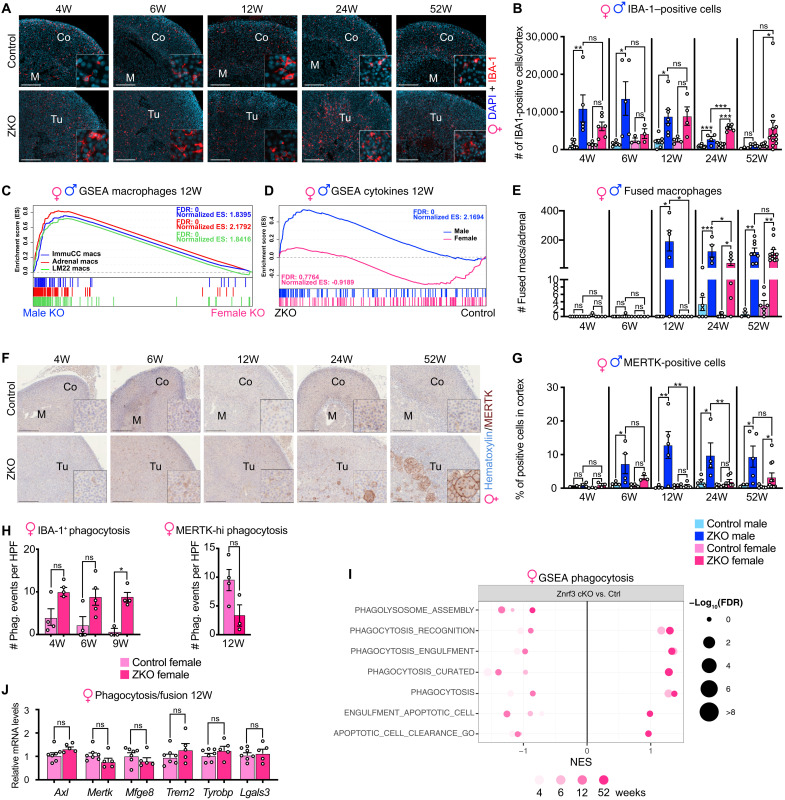
Recruitment of phagocytic macrophages is delayed in females. (**A**) IHC for IBA-1 in female control and *Znrf3 cKO* adrenals from 4 to 52 weeks. (**B**) Quantification of the IBA-1 index as the ratio of IBA-1^+^ cells over total nuclei in the cortex of control and *Znrf3 cKO* male (blue) and female (pink) mice. (**C**) GSEA of macrophage gene sets in male *Znrf3 cKO* compared with female *Znrf3 cKO* adrenals at 12 weeks. (**D**) GSEA of the cytokine gene set in *Znrf3 cKO* males and females compared with their respective control adrenals at 12 weeks. (**E**) Number of fused macrophages (at least two nuclei) in control and *Znrf3 cKO* male and female adrenals. (**F**) IHC for MERTK in control and *Znrf3 cKO* female adrenals. (**G**) Quantification of the MERTK^+^ index as the ratio of MERTK-positive cells over total nuclei in the cortex of control and *Znrf3 cKO* male (blue) and female (pink) mice. (**H**) Quantification of phagocytic events following IHC for IBA-1 and 3βHSD (IBA-1^+^ phagocytosis) or MERTK and SF-1 (MERTK^high^ phagocytosis) in control and *Znrf3 cKO* females. Quantification was performed on 10 HPFs (×40) per individual mouse. (**I**) GSEA of phagocytosis/efferocytosis gene sets in *Znrf3 cKO* female adrenals compared with controls. (**J**) RT-qPCR analysis of the expression of phagocytosis- and macrophage fusion–associated genes in control and *Znrf3 cKO* female adrenals at 12 weeks. Scale bars, 200 μm. Graphs represent means ± SEM. Statistical analyses were conducted by two-way ANOVA in (B), (E), and (G) and by Mann-Whitney tests in (H) and (J). *P < 0.05; **P < 0.01; ***P < 0.001; ****P < 0.0001.

In contrast with males, female *Znrf3 cKO* adrenals progress from hyperplasia at 4 weeks to development of full-fledged metastatic carcinomas at 78 weeks (Fig. 1). Analysis of the overall mononuclear macrophage population by IHC for IBA-1 showed increased recruitment of IBA-1+ macrophages in *Znrf3 cKO* females from 4 to 52 weeks (Fig. 4A). However, counting of IBA-1+ cells suggested that macrophage recruitment was milder than in males from 4 to 12 weeks (Fig. 4, A nd B). This was confirmed by GSEA (Fig. 4C), showing a robust enrichment in macrophage signatures in male KOs compared with female KOs at 12 weeks and RT-qPCR analyses of *Cd68*, *Adgre1*, and *Cd11b* (fig. S5A). CIBERSORTx analysis also failed to show macrophage enrichment, although there was up-regulation of monocytes at 12 weeks (fig. S5B). Consistent with observations in male adrenals, flow cytometry analyses showed that most macrophages that were present in both control and *Znrf3 cKO* females displayed mixed M1 and M2 features at 6 weeks (fig. S5C). Milder inflammatory response in female KOs was also confirmed by the absence of cytokine signature enrichment at 12 weeks, compared with male KOs (Fig. 4D). By 24 and up to 52 weeks, the number of IBA-1+ cells significantly increased in female *Znrf3 cKO* adrenals, which was accompanied by a mild but significant increase in mRNA accumulation of *Adgre1* at 24 weeks and *Cd68* at 52 weeks (Fig. 4, A and B, and fig. S5A). However, this was still not associated with enrichment of cytokines (fig. S5D). Together, this showed that macrophage recruitment was delayed in female *Znrf3 cKO* adrenals and was not associated with robust inflammation. In males, regression of hyperplasia is associated with fusion of mononuclear macrophages to form MGCs (Fig. 3). Whereas fused macrophages were already present in large numbers in 12 weeks *Znrf3 cKO* males, they did not appear before 24 weeks in females (Fig. 4E). Consistent with delayed fusion, fused macrophages harbored less nuclei (fig. S5E) and were smaller than in males at this stage (fig. S5F). In male *Znrf3 cKO* adrenals, acquisition of high phagocytic capacities is associated with infiltration of MERTK^high^ macrophages as early as 6 weeks (Figs. 3D and 4G). In contrast, these were scarce until 24 weeks in female *Znrf3 cKO* adrenals (Fig. 4, F and G). They mostly represented fused macrophages (Fig. 4F) and were only significantly increased in numbers at 52 weeks (Fig. 4G). This suggested that phagocytosis of hyperplastic mutant cells may be impaired in female KOs. Although there was trend for increased phagocytosis by IBA-1^+^ macrophages, it only reached significance at 9 weeks (Fig. 4H). Furthermore, the rate of phagocytosis was much lower than in males, barely reaching 10 events per high-power field in female KOs, compared with more than 40 in male KOs (Fig. 3E). The low phagocytic capacity in females was even more evident when analyzed within MERTK^high^ macrophages at 12 weeks (Fig. 4H). This was supported by the lack of enrichment of phagocytosis-related gene signatures at any time point (Fig. 4I), which was further confirmed by RT-qPCR at 12 weeks (Fig. 4J). Together, these data strongly suggest that delayed recruitment and impaired function of phagocytic macrophages allow progression of hyperplasia in *Znrf3 cKO* females.

### Androgens are sufficient to trigger early recruitment of phagocytic macrophages and regression of hyperplasia

**Fig. 5.**
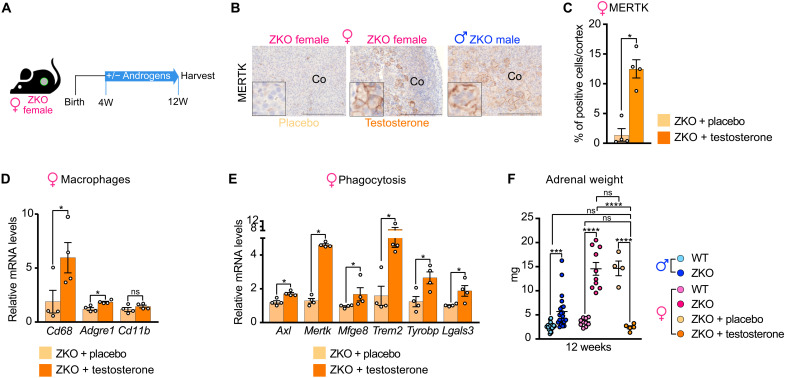
Androgens are sufficient to trigger early recruitment of phagocytic macrophages and regression of hyperplasia. (**A**) Cartoon of the experimental setup. (**B**) IHC for MERTK in 12-week-old placebo- and testosterone-treated *Znrf3 cKO* females. An untreated 12-week-old *Znrf3 cKO* male was included as a reference. (**C**) Quantification of the MERTK^+^ index as the ratio of MERTK^+^ cells over total nuclei in the cortex of placebo- and testosterone-treated females at 12 weeks. (**D**) RT-qPCR analysis of the expression of macrophage-related genes in placebo- and testosterone-treated *Znrf3 cKO* female adrenals at 12 weeks. (**E**) RT-qPCR analysis of the expression of phagocytosis- and macrophage fusion–associated genes in placebo- and testosterone-treated *Znrf3 cKO* female adrenals at 12 weeks. (**F**) Adrenal weights from placebo- and testosterone-treated 12-week-old *Znrf3 cKO* females. Twelve-week-old untreated control males/females and *Znrf3 cKO* males/females from Fig. 1 (A and E) were included as a reference. Scale bar, 200 μm. Graphs represent means ± SEM. Statistical analyses in (C) to (E) were conducted by Mann-Whitney tests. In (F), statistical analyses were conducted by two-way ANOVA, followed by a Kruskal-Wallis post hoc test. *P < 0.05; **P < 0.01; ***P< 0.001; ****P < 0.0001.

Sexually dimorphic phenotypic differences in phagocytic macrophage recruitment and regression of hyperplasia occur between 6 and 12 weeks, which coincides with onset of puberty in mice. To evaluate a potential contribution of androgens to this phenomenon, *Znrf3 cKO* females were implanted with placebo or testosterone pellets from 4 to 12 weeks and their adrenals were then harvested (Fig. 5A). As expected, placebo-treated female adrenals were almost completely devoid of MERTK^high^ macrophages (Fig. 5, B and C). In sharp contrast, testosterone-treated females displayed abundant infiltration of both mononuclear and fused MERTK^high^ macrophages, which was almost equivalent to 12-week-old males (Fig. 5, B and C). Infiltration of macrophages was further confirmed by RT-qPCR, showing increased expression of *Cd68* and *Adgre1*, following androgen treatment (Fig. 5D). RT-qPCR analysis of phagocytosis-associated gene expression also showed increased accumulation of *Axl*, *Mertk*, *Mfge8*, *Trem2*, *Tyrobp*, and *Lgals3*, suggesting that testosterone treatment stimulated recruitment of phagocytic macrophages (Fig. 5E). Consistent with this hypothesis, testosterone treatment was associated with a marked decrease in *Znrf3 cKO* female adrenal weight, which returned to control levels (Fig. 5F). Together, these experiments show that androgens are sufficient to induce recruitment of phagocytic macrophages, which results in regression of hyperplasia.

### Recruitment of phagocytic macrophages in male *Znrf3 cKO* mice is associated with sexually dimorphic induction of senescence

**Fig. 6.**
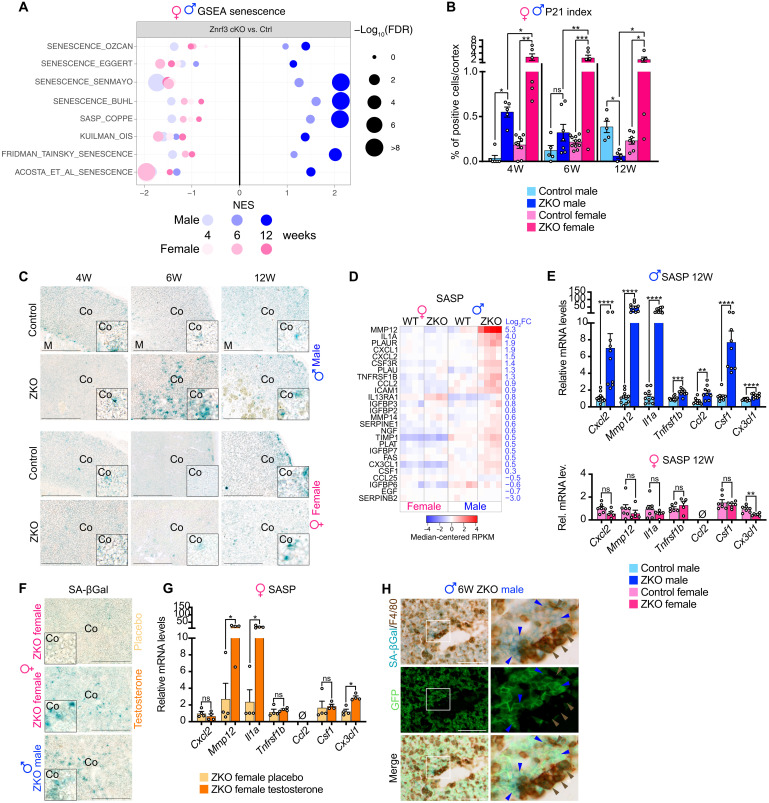
Recruitment of phagocytic macrophages in male *Znrf3 cKO* mice is associated with sexually dimorphic induction of senescence. (**A**) GSEA of senescence-associated gene sets in *Znrf3 cKO* male and female adrenals compared with their respective controls. (**B**) Quantification of the p21^+^ index as the ratio of p21-positive cells over total nuclei in the cortex of male and female control and *Znrf3 cKO* mice from 4 to 12 weeks. (**C**) Detection of the SA-βGal activity on frozen tissue sections from male and female control and ZKO at 4, 6, and 12 weeks. (**D**) Expression of SASP genes in 12-week-old male and female control and *Znrf3 cKO* adrenals. Genes were selected on the basis of significant deregulation in 12-week-old male *Znrf3 cKO* adrenals (FDR < 0.1) and sorted by log_2_ fold change (log_2_FC). (**E**) RT-qPCR analysis of SASP gene expression in control and *Znrf3 cKO* males (top) and females (bottom). (**F**) Detection of SA-βGal activity in the adrenals of *Znrf3 cKO* females that received placebo or testosterone treatment from 4 to 5 weeks. An untreated 6-week-old *Znrf3 cKO* male was included as a reference. (**G**) RT-qPCR analysis of SASP gene expression in placebo- and testosterone-treated *Znrf3 cKO* females from Fig. 5. (**H**) IHC for GFP (marking *SF-1:Cre*–mediated recombination of mTmG in steroidogenic cells), F4/80, and SA-βGal activity. Right panels show a high-magnification crop of the area delineated in white in left panels. Blue arrowheads show senescent GFP^+^ cells; brown arrowheads show F4/80^+^ macrophages. Scale bars, 200 μm (C to F) and 100 μm (H). Graphs represent means ± SEM. Statistical analyses were conducted by two-way ANOVA in (B) and by Mann-Whitney tests in (E). *P < 0.05; **P < 0.01; ***P < 0.001; ****P < 0.0001.

Recruitment of myeloid cells to preneoplastic lesions has been associated with induction of senescence (*36*, *37*). To evaluate a potential role of senescence in the sexually dimorphic recruitment of phagocytes in the adrenal cortex of *Znrf3 cKO* mice, we evaluated enrichment of senescence-associated signatures in males and females from 4 to 12 weeks. Whereas most of these signatures were significantly enriched in *Znrf3 cKO* males at 6 and 12 weeks, there was no or negative enrichment in females (Fig. 6A). This suggested that ablation of *Znrf3* resulted in male-specific induction of senescence. To further evaluate this hypothesis, we first analyzed expression of the cell cycle inhibitor p21. In these experiments, steroidogenic cells were labeled by green fluorescent protein (GFP), which was expressed by the mTmG locus following *SF-1:Cre*–mediated recombination. Consistent with induction of senescence, there was a significant increase in p21 labeling index within GFP^+^ steroidogenic cells in *Znrf3 cKO* males at 4 weeks (Fig. 6B and fig. S6, A and B). Levels of p21^+^ cell accumulation returned to normal at 6 weeks in *Znrf3 cKO* males and were significantly reduced at 12 weeks, consistent with phagocytosis of senescent cells (Fig. 6B). Unexpectedly, a significant increase in p21 labeling was also observed in *Znrf3 cKO* females at 4 weeks and maintained up to 12 weeks (Fig. 6B and fig. S6B), suggesting that cell cycle was arrested in both males and females, following *Znrf3* ablation. To further assess induction of senescence, we analyzed activity of the prototypic senescence-associated acidic β-galactosidase (SA-βGal). This showed that a few cells were found in the subcapsular area and at the cortical-medullary junction in control males and females, which was further increased in control females at 12 weeks. This suggested that spontaneous senescence was taking place in these regions (Fig. 6C). Notably, SA-βGal staining was increased within the inner cortex of male *Znrf3 cKO* mice at 6 weeks and to a lesser extent at 12 weeks, consistent with phagocytic clearance of senescent cells in male adrenals (Fig. 6C). In contrast, there was no increase in SA-βGal staining in *Znrf3 cKO* females, which displayed a similar pattern to controls (Fig. 6C). This suggested that although proliferation was arrested in both males and females, senescence was only induced in male *Znrf3 cKO* adrenals. To further confirm this, we evaluated expression of a senescence-associated secretory phenotype (SASP) in our RNA sequencing data. This analysis showed that the 26 SASP-coding genes that were significantly deregulated in 12-week-old *Znrf3 cKO* male adrenals were not deregulated in females (Fig. 6D), suggesting that establishment of a SASP was male specific. This was further confirmed by male-specific enrichment of gene sets for nuclear factor κB (NFκB) signaling, which plays a key role in SASP induction (fig. S6C) (*49*, *50*). RT-qPCR analyses confirmed significant up-regulation of *Mmp12* and *Il1a* at 6 weeks and of *Cxcl2*, *Mmp12*, *Il1a*, and *Tnfrsf1b* at 12 weeks in male but not female adrenals (Fig. 6E and fig. S6D). Among SASP factors, the three monocyte/macrophage chemoattractants *Ccl2*, *Csf1*, and *Cx3cl1* were up-regulated in male adrenals and not deregulated (*Csf1*), undetectable (*Ccl2*), or even down-regulated in females (*Cx3cl1*) (Fig. 6E and fig. S6D). *Znrf3 cKO* female mice that received testosterone (Fig. 5) also showed induction of SA-βGal after 1 week of treatment (from 4 to 5 weeks; Fig. 6F). This was associated with up-regulation of *Mmp12*, *Il1a*, and the chemoattractant *Cx3cl1*, but not *Ccl2* (undetectable) or *Csf1* (Fig. 6G). This suggested that testosterone played a key role in senescence induction, which, in turn, allowed recruitment of macrophages through SASP factors, including CX3CL1. Consistent with this hypothesis, F4/80-positive macrophages were found in very close proximity to SA-βGal– and GFP-positive steroidogenic cells in the adrenal cortex of male *Znrf3 cKO* mice at 6 weeks (Fig. 6H). In situ RNA hybridization analyses further showed increased accumulation of *Cx3cl1* transcripts in cells surrounding fused macrophages in male *Znrf3 cKO* and androgen-treated but not placebo-treated female *Znrf3 cKO* adrenals at 12 weeks (fig. S6E). Together, these data strongly suggest that male-specific androgen-driven induction of senescence and SASP results in recruitment, activation, and fusion of highly efficient phagocytes that prevent tumor progression in male *Znrf3 cKO* mice.

### Aggressive tumorigenesis is associated with infiltration of nonphagocytic macrophages in female adrenals

**Fig. 7.**
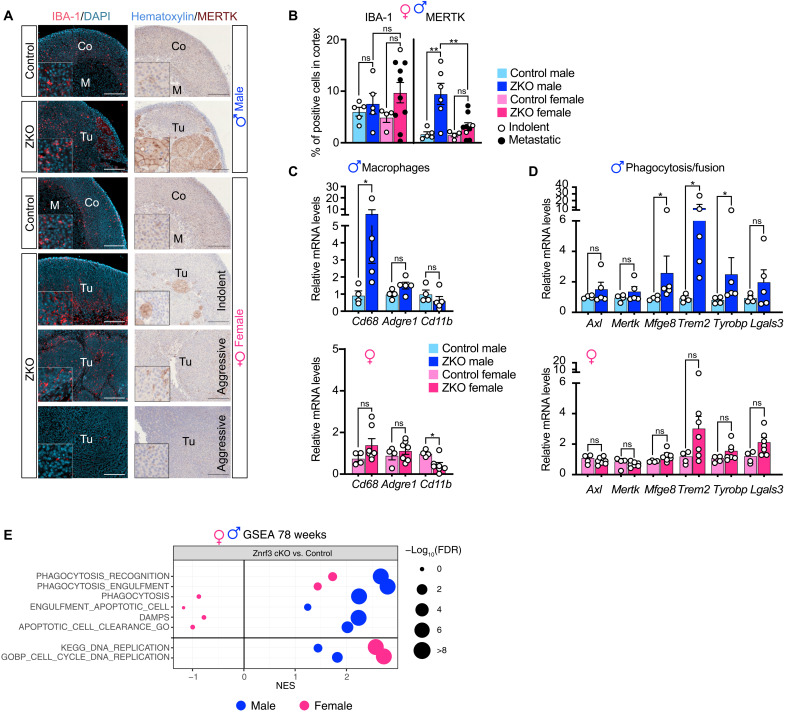
Aggressive tumorigenesis is associated with infiltration of nonphagocytic macrophages in female adrenals. (**A**) IHC for IBA-1 and MERTK in control and *Znrf3 cKO* males/females at 78 weeks. For *Znrf3 cKO* females, the panels represent indolent tumors (no metastases) and aggressive tumors with or without macrophage infiltration. (**B**) Quantification of the IBA-1^+^ and MERTK^+^ index as the ratio of IBA-1–positive (left) or MERTK-positive (right) cells over total nuclei in the cortex of male and female control and *Znrf3 cKO* mice at 78 weeks. Values for primary tumors associated with metastases are shown as black dots. (**C**) RT-qPCR analysis of the expression of macrophage-related genes in control and *Znrf3 cKO* males (top) and control and *Znrf3 cKO* females (bottom) at 78 weeks. (**D**) RT-qPCR analysis of the expression of phagocytosis- and macrophage fusion–associated genes in control and *Znrf3 cKO* males (top) and control and *Znrf3 cKO* females (bottom) at 78 weeks. (**E**) GSEA of gene expression data (RNA sequencing) from 78-week-old control and *Znrf3 cKO* male and female adrenals. The plot represents enrichment of phagocytosis-associated gene sets and DNA replication–associated gene sets in *Znrf3 cKO* compared with controls (sex-matched). Scale bars, 200 μm. Graphs represent means ± SEM. Statistical analyses were conducted by two-way ANOVA in (B) and Mann-Whitney tests in (E). *P < 0.05; **P < 0.01.

To further gain insight into the role of macrophages at late stages of tumorigenesis, we evaluated infiltration of macrophages in 78-week-old adrenal lesions in both male and female mice. At this stage, male *Znrf3 cKO* adrenals were still infiltrated by IBA-1+ macrophages that were scattered throughout the cortex (Fig. 7A). However, quantification of the IBA-1 index showed that in contrast with earlier stages, infiltration was equivalent to control males (Fig. 7B). In female *Znrf3 cKO*, IBA-1+ infiltration was somewhat heterogeneous within the tissue, with areas of high infiltration and zones that were almost devoid of macrophages (Fig. 7A). There was also interindividual heterogeneity. Some tumors were still infiltrated at levels comparable to controls, whereas others showed much less IBA-1^+^ cells or virtually no macrophages (Fig. 7, A and B). There was no overall difference between indolent and aggressive (metastatic) tumors with respect to IBA-1^+^ index (Fig. 7B). However, macrophage exclusion was observed in a subset of 2 of 10 aggressive tumors (Fig. 7, A and B). Consistent with IHC analyses, accumulation of mRNA encoding macrophage markers was unaltered (*Cd68* and *Adgre1*) or decreased (*Cd11b*) in female *Znrf3 cKO* compared to controls (Fig. 7C). Although accumulation of *Adgre1* and *Cd11b* mRNA was unaltered, *Cd68* was still strongly accumulated in male *Znrf3 cKO* adrenals (Fig. 7C). Because we showed high expression of CD68 in fused macrophages at earlier stages (Fig. 2G), this suggested that active phagocytes may still be accumulating in male KO adrenals at 78 weeks. Consistent with this idea, there were still large numbers of MERTK^high^ fused macrophages in 78-week-old *Znrf3 cKO* male adrenals (Fig. 7, A and B), which was correlated with overexpression of *Mfge8*, *Trem2*, and *Tyrobp* in RT-qPCR (Fig. 7D). In contrast, female *Znrf3 cKO* adrenals showed scarce infiltration of MERTK^high^ fused macrophages, although a few of them could still be observed in indolent tumors (Fig. 7, A and B). Consistent, with these observations, there was no deregulation of phagocytosis/fusion markers in *Znrf3 cKO* female adrenals at this stage (Fig. 7D). GSEA showed strong enrichment of phagocytosis-associated signatures but no enrichment of DNA proliferation/cell cycle pathways in male *Znrf3 cKO* RNA sequencing data at 78 weeks (Fig. 7E). In contrast, female KOs showed high enrichment of proliferation signatures but no enrichment of phagocytosis (Fig. 7E). Together, these data strongly suggest that although macrophages are still present within tumor tissues at 78 weeks in both males and females, the lack of phagocytic activity is associated with aggressive tumor progression in females.

### Phagocytic macrophage signatures are prominent in male ACC patients and associated with better prognosis

**Fig. 8.**
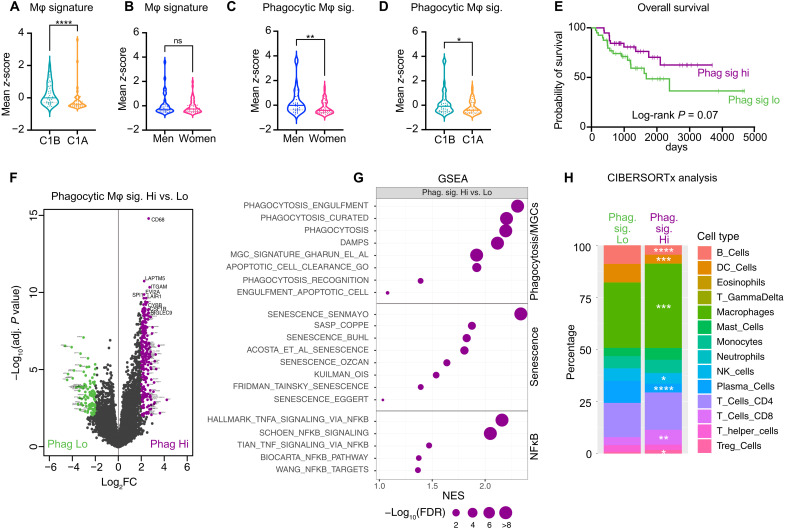
Phagocytic macrophage signatures are prominent in male ACC patients and associated with better prognosis. (**A**) Expression of a global macrophage gene signature in ACC patients from the TCGA program, dichotomized as patients with good (C1B) and poor (C1A) prognosis. (**B**) Expression of a global macrophage gene signature in ACC patients from the TCGA program, dichotomized as men and women. (**C**) Expression of a phagocytic macrophage gene signature in ACC patients from the TCGA program, dichotomized as men and women. (**D**) Expression of a phagocytic macrophage gene signature in ACC patients from the TCGA program, dichotomized as patients with good (C1B) and poor (C1A) prognosis. (**E**) Survival analysis of patients of the TCGA program, dichotomized as patients with high (purple) or low (green) expression of the phagocytic signature. (**F**) Volcano plot displaying differential gene expression between patients with high and low expression of the phagocytic signature. Purple dots represent genes with log_2_ fold change > 2 and FDR < 0.01. Green dots represent genes with log_2_ fold change ≤ 2 and FDR < 0.01. (**G**) GSEA of phagocytosis, senescence, and NFκB-associated gene sets in patients with high expression of the phagocytic signature compared with patients with low expression of the signature. (**H**) Stacked bar plots representing immune cell populations deconvoluted using CIBERSORTx and the LM22 expression matrix from gene expression data in ACC patients with low and high phagocytic signatures. Statistical analyses in (A) to (D) were conducted by Mann-Whitney tests. *P < 0.05; **P < 0.01; ****P < 0.0001.

To further evaluate the role of macrophages in ACC progression, we evaluated their infiltration within human ACC. For this, we used RNA sequencing data from the TCGA consortium (79 sequenced ACCs) and evaluated expression of a 10-gene signature [based on single-cell RNA sequencing (scRNA-seq) data from mouse adrenals (*51*)] as a proxy to general macrophage infiltration. Tumors of the good prognosis group, defined as C1B (*27*), had significantly higher expression of the macrophage signature than tumors of the bad prognosis C1A group (Fig. 8A). Consistent with our data showing similar infiltration of IBA-1^+^ macrophages in male and female *Znrf3 cKO* adrenals at 78 weeks (Fig. 7B), there was no difference in the general macrophage signature expression between ACC in men (*n* = 31) and women (*n* = 48) (Fig. 8B). However, a three-gene phagocytic macrophage signature (*CD68*, *TREM2*, and *TYROBP*) was significantly expressed at higher levels in men (Fig. 8C) and in the C1B group of ACC with favorable prognosis (Fig. 8D). In contrast with the global macrophage signature (fig. S7A), high expression of the phagocytic signature (above median) was associated with better survival, compared with low expression (below median) in Kaplan-Meier analysis (Fig. 8E). Of note, the two signatures were not associated with hormonal status of patients (fig. S7B). Together, this suggested that infiltration with phagocytic macrophages was more frequent in men than in women and was associated with better prognosis.

Detailed analysis of RNA sequencing data identified 365 genes that were significantly deregulated [FDR < 0.01, abs(Log2FC > 2)] between the groups of high and low expression of the phagocytic signature (Fig. 8F). As expected, macrophage-associated genes such as *CD68*, *CSF1R*, *ITGAM*, *LAPTM5*, *CYBB*, and *SIGLEC9* were up-regulated in phagocytic-high patients (Fig. 8F). GSEA confirmed enrichment of macrophages (fig. S7C) and phagocytosis signatures (Fig. 8G). Consistent with data in our mouse models, phagocytic signatures were also associated with enrichment of senescence and NFκB signaling gene sets (Fig. 8G), suggesting that these pathways may also play a role in phagocytic macrophage recruitment in ACC patients. GO analysis using the C5 GO database (MSigDB) showed that the top 35 positively enriched gene sets were all related with immune response and inflammation in patients with high expression of the phagocytic signature, suggesting that this subgroup was mounting a more profound immune response than patients with low expression of the signature (fig. S7D). Deconvolution of RNA sequencing data using CIBERSORTx showed that macrophages were the most prominent immune cell population in the two groups of patients, consistent with mouse adrenals (Fig. 8H). It also showed that enrichment of macrophage signatures in the phagocytic-high subgroup of patients was associated with increased cytotoxic CD8^+^ T lymphocyte signatures (Fig. 8H). However, this was also correlated with lower B cells, plasma cells, and natural killer (NK) cells and higher T regulatory cell infiltration (Fig. 8H), suggesting that the phagocytic-high subgroup of patients had a broad alteration of the immune tumor microenvironment. Together, these observations suggest that phagocytic macrophages, which are more prominent in male than in female ACC patients, are associated with senescence, global innate and adaptive immune response, and better prognosis.

## DISCUSSION

**Fig. 9.**
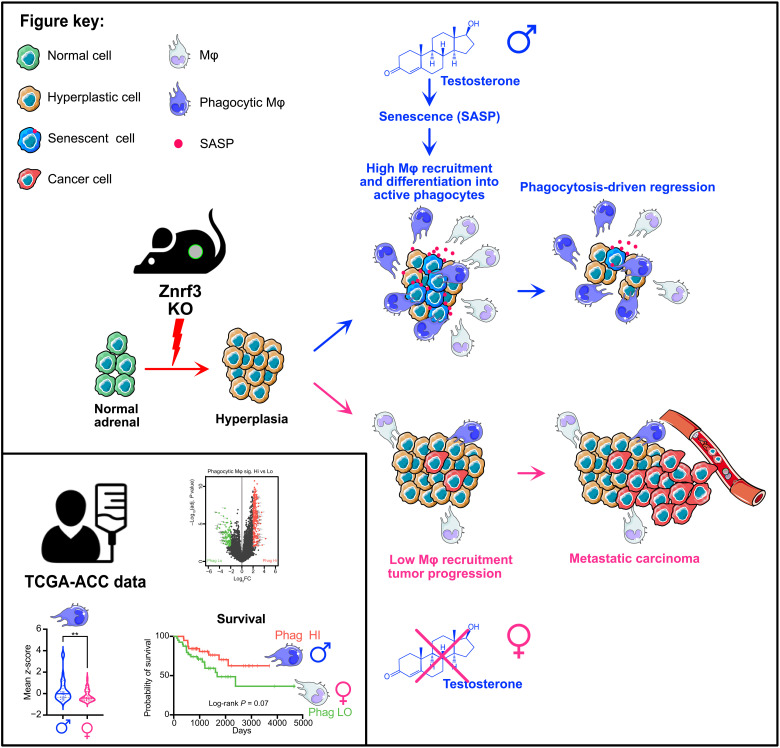
Graphical abstract. Inactivation of *Znrf3* in steroidogenic cells of the mouse adrenal cortex is associated with sexually dimorphic tumor progression. Although female KOs develop metastatic carcinomas at 18 months, adrenal hyperplasia regresses in male KOs. This male-specific phenotype is associated with androgen-dependent induction of senescence, recruitment, and differentiation of highly phagocytic macrophages that clear out senescent cells. In contrast, in females, macrophage recruitment is delayed and dampened, which allows for aggressive tumor progression. Analysis of TCGA-ACC data shows that phagocytic macrophages are more prominent in men and associated with better prognosis. Together, these data show that phagocytic macrophages are key players in the sexual dimorphism of ACC and establish them as previously unidentified allies in the fight against this cancer.

Apart from reproductive tissues, cancers are generally more frequent and aggressive in men than in women, even after adjusting for known risk factors (*1*, *2*). Although ACC is one of the rare exceptions to this rule, the mechanisms underlying the higher incidence and aggressiveness in women remain elusive. Here, we show that conditional deletion of *Znrf3* within steroidogenic cells of the adrenal cortex results in sexually dimorphic development of full-fledged metastatic ACC in female mice over an 18-month time course, whereas the initial hyperplasia gradually regresses in males (Fig. 9). By a combination of RNA sequencing, flow cytometry, and IHC analyses, we show that *Znrf3 cKO* males efficiently recruit macrophages from early stages of preneoplastic transformation, following induction of senescence. We further show that these macrophages, which differentiate as potent phagocytes, are required for clearance of preneoplastic cells. Although females also mount an innate immune response to preneoplastic transformation, it is delayed compared to males and never achieves efficient clearance of preneoplastic cells. This phenomenon is maintained up to 78 weeks, when indolent lesions in male *Znrf3 cKO* adrenals are still infiltrated with large amounts of phagocytic macrophages, as opposed to aggressive female tumors (Fig. 9). Consistent with our findings in mice, we show that a phagocytic macrophage signature is more prominent in male than in female ACC patients, where it is associated with better prognosis (Fig. 9). This strongly suggests that the sexual dimorphism of ACC may result from differential recruitment and activation of phagocytic TAMs, which prevent both tumor initiation and progression in the adrenal cortex.

This is in contrast with most data of the literature showing that TAMs are generally associated with tumor progression and poor prognosis in many cancers, although they may initially prevent tumor initiation (*36*, *37*, *52*–*54*). Plasticity and diversity of TAMs explain their divergent functions. The standard dual classification of macrophages postulates that M1 macrophages that differentiate in response to proinflammatory cytokines (e.g., interferons and tumor necrosis factors) are involved in antitumor activities, whereas M2 macrophages that differentiate in response to immunomodulatory signals [e.g., IL-4, IL-10, and transforming growth factor–β (TGF-β)] are associated with tumor promotion (*55*). However, recent scRNA sequencing analyses of tumor-infiltrating myeloid cells showed that M1 and M2 gene signatures were coexpressed in macrophage subsets from almost all cancer types (*53*). Consistent with this idea, our RNA sequencing and flow cytometry analyses suggested that macrophages that accumulate in the adrenals of *Znrf3 cKO* males had mixed characteristics of the M1 and M2 phenotypes. Furthermore, we did not find evidence of overexpression of canonical tumoricidal macrophage markers such as the proinflammatory cytokines IL-1β, IL-2, IL-6, IL-12, and IL-23 (Fig. 2B) or inducible nitric oxide synthase (iNOS), which metabolizes arginine into the killer molecule nitric oxide. This suggests that the tumoricidal function of adrenal macrophages relies on alternative activities. Consistent with this idea, we show a very strong increase in the phagocytic activity of macrophages in *Znrf3 cKO* male adrenals compared with their wild-type littermates and *Znrf3 cKO* females. This activity is associated with cytoplasmic accumulation of CD68 and high membrane expression of MERTK, TYROBP, and TREM2, which play a central role in the phagocytic process (*40*, *43*, *45*–*47*). Although *MERTK* expression did not correlate with the presence of macrophages in ACC patients, we show that increased expression of the phagocytic *CD68/TREM2/TYROBP* signature is correlated with better prognosis, within the TCGA cohort. This strongly suggests that phagocytosis plays a central role in the tumoricidal activity of macrophages in ACC. scRNA-seq in human and mouse colorectal cancer identified a population of C1QC^+^ TAMs, characterized by high levels of *C1QA/B/C*, *TREM2*, and *MERTK* expression, which were associated with potential recruitment and activation of T cells, phagocytosis, and better prognosis (*53*, *56*). Although we did not analyze macrophages by scRNA-seq in *Znrf3 cKO* adrenals, our bulk RNA sequencing data show strong up-regulation of all these markers (Fig. 3B), which are mostly expressed by macrophages in scRNA-seq datasets from wild-type mouse adrenals (fig. S8A). This strongly suggests that tumoricidal TAMs found in ACC may be related with the phagocytic C1QC^+^ TAMs identified in other cancers (*53*, *56*).

A very notable feature of the phenotype is the strong sexual dimorphism in immune response to neoplasia, due to early recruitment of tumoricidal phagocytic macrophages, specifically in male mice. We further show that testosterone treatment of females from 4 to 12 weeks is sufficient to trigger a response, which is comparable to males and results in regression of hyperplasia (Fig. 5). This strongly suggests a role of male hormones in this phenomenon and raises the question of the underlying mechanisms. One possibility is an intrinsic sexual dimorphism of macrophages within the adrenal, which would result in differential responses to oncogenic transformation of steroidogenic cells. Recruitment, replenishment, and activation mechanisms of macrophages and other immune cell types have been shown to diverge between males and females, resulting in sexually dimorphic responses to infection and proinflammatory stimuli. However, in most instances, female macrophages are more responsive to stimuli, mount a more robust response, and have higher phagocytic capacities than male macrophages (*57*–*63*). This suggests that the stronger inflammatory response observed in male *Znrf3 cKO* adrenals may result from indirect effects of sex hormones. Consistent with this, single-cell sequencing data suggest that the androgen receptor *Ar* is only expressed in a small subset of adrenal macrophages, characterized by lower expression of *Trem2* and *Mertk,* which is unlikely to represent the major population of macrophages in *Znrf3 cKO* adrenals (fig. S8A). Our data showing a strong association between induction of SASP and recruitment of macrophages suggest that androgens may stimulate the tumoricidal response by inducing release of senescence-associated cytokines by *Znrf3 cKO* cells (Fig. 6). In line with this hypothesis, androgen receptor (AR) activation was shown to induce p53-independent senescence in prostate cancer cells (*64*, *65*), and a short-term testosterone treatment was sufficient to induce SA-βGal activity in female *Znrf3 cKO* adrenals (Fig. 6). This raises the question of the links between *Znrf3* inactivation, AR signaling, and senescence induction. One may speculate that the recently documented sexual dimorphism in cortical cell proliferation, renewal, and progenitor populations (*8*, *9*) may result in sexually dimorphic response to *Znrf3* inactivation. In this context, the hyperproliferation observed in both male and female *Znrf3 cKO* adrenals may result in faster exhaustion of progenitor pools in males and subsequent induction of senescence. However, the rapid induction of SA-βGal in testosterone-treated *Znrf3 cKO* females suggests that this is an unlikely scenario. Alternatively, these findings may reflect a previously unknown function of ZNRF3 in the control of cellular homeostasis. Although we were able to show a mild induction of *Axin2* accumulation in *Znrf3 cKO* adrenals by RNA in situ hybridization (*30*), analysis of our RNA sequencing data did not show evidence of canonical WNT signaling induction in either male or female KOs (fig. S8B), when compared with a previously published model of constitutive β-catenin activation (*66*). This suggests that the impact of *Znrf3* inactivation on senescence induction may involve WNT-independent mechanisms.

We could find large numbers of IBA-1^+^ macrophages (up to 15% of total cells in the tumor) in aggressive tumors in 78-week-old *Znrf3 cKO* female mice (Fig. 7B), although they were not differentiated as MERTK^high^ active phagocytes. The presence of macrophages was further confirmed in ACC patients, where CIBERSORTx deconvolution suggested that they represented 31% of all immune cells, even in the tumors expressing low levels of the phagocytic signature (40% in phagocytic-high tumors; Fig. 8H). These data suggest that even in aggressive phagocytic-low lesions, macrophages may be reprogrammed to stimulate their tumoricidal potential. Although most current macrophage-related therapies aim to deplete this cell type in tumors, more recent strategies that stimulate tumoricidal activity and particularly phagocytosis of tumor cells by macrophages are currently being investigated (*55*). These include approaches that aim at inhibiting the CD47 “don’t-eat-me” signal produced by cancer cells and/or the SIRPα receptor for CD47 on macrophages, as well as stimulation of Toll-like receptor (TLR) signaling with TLR agonists (*55*). One important factor that these therapeutic approaches will have to consider in the context of ACC is the presence of high levels of glucocorticoids produced by adrenal steroidogenic cells, in particular within hormonally active tumors. Although glucocorticoids do not have the same detrimental impact on macrophages that they have on lymphocytes, they are generally associated with M2-like tolerogenic differentiation (*67*). Therefore, therapeutics targeting macrophages in ACC should probably consider combining macrophage activation with inhibition of glucocorticoid production or signaling, which would also favor recruitment of adaptive immune cells to the lesion. Availability of our clinically relevant mouse model will allow evaluation of these innovative options.

A recently posted BioRxiv preprint reports similar findings in a mouse model of *Znrf3* inactivation (*68*). Although differences in genetic backgrounds resulted in a more pronounced immune response in females in their model than in ours, Warde and colleagues also showed that male *Znrf3* KO adrenals mounted a profound macrophage-dependent antitumor response, further strengthening the findings reported here.

In conclusion, we describe a previously unidentified interaction between tumor suppressor inactivation, senescence induction, and recruitment of tumoricidal macrophages, which results in sexually dimorphic adrenal cancer development. This provides unanticipated insight into the strong gender bias of this particularly aggressive cancer and may help develop innovative macrophage-centric therapeutic approaches.

## MATERIALS AND METHODS

### Mice

All experiments with mice were in accordance with protocols approved by the Auvergne Ethics Committee [Autorisation de Projet utilisant des Animaux à des Fins Scientifiques (APAFIS) #27623-2021021611362535 v1]. They were conducted in agreement with international standards for animal welfare to minimize animal suffering. ZKO were generated by mating *Znrf3^fl/fl^* mice (*28*) with *SF1-Cre^high^* mice (*69*). In experiments that did not involve flow cytometry, the mTmG reporter gene was also included in the breeding scheme (*70*). Mice were bred and maintained on a C57Bl/6 genetic background. Mice were euthanized by decapitation at the end of experiments, and blood was collected in vacuum blood collection tubes (VFD053STK, Terumo). Adrenals were extracted, cleaned of excess fat, weighed, and immediately fixed in 4% paraformaldehyde or stored at −80°C. Littermate control animals were used in all experiments.

### Immunohistology

Adrenals were fixed in 4% paraformaldehyde overnight at 4°C and then washed two times in phosphate-buffered saline (PBS). For the paraffin embedding, adrenals were dehydrated through an ethanol gradient. Then, they were incubated for 2 hours in Histoclear (HS200, National Diagnostics, Fisher Scientific, Illkirch, France) and embedded in paraffin. For frozen sections, adrenals were successively placed in 10 and 15% PBS-sucrose solutions for 20 min, then in 20% PBS-sucrose solution for 1 hour, and in 50:50 optimal cutting temperature (OCT)–sucrose 20% solution overnight. Last, they were embedded in pure OCT solution and stored at −80°C. Paraffin and OCT samples were cut into 5- and 10-μm sections, respectively. H&E staining was performed with a Microm HMS70 automated processor (Microm Microtech, Francheville, France), according to standard procedures. Antibody information, dilutions, and unmasking conditions are listed in table S1. Notably, the TREM2 antibody (*71*) was supplied from the Haass laboratory at Ludwig Maximilians University Munich. After deparaffinization with Histoclear and rehydration in decreasing ethanol gradients, unmasking was performed by boiling slides for 20 min in the appropriate unmasking solution. Next, endogenous peroxidases were inactivated by incubating slides with 0.3% hydrogen peroxide for 30 min at room temperature. After blocking for 1 hour, slides were incubated overnight at room temperature with primary antibodies at the indicated concentrations (table S1). Primary antibodies were detected with appropriate species polymers (ImmPress Polymer Detection Kit, Vector Laboratories). Polymer-coupled horseradish peroxidase (HRP) activity was then detected with either NovaRED (SK-4800, Vector Laboratories) for bright-field images or tyramide signal amplification (TSA)-Alexa–coupled fluorochromes for fluorescence (Thermo Fisher Scientific, Alexa_488 B40953, Alexa_555 B40955, and Alexa_647 B40958). For double-IHC experiments, HRP was inactivated by incubation with 0.02% HCl for 20 min after detection of the first antibody to avoid cross-reaction. Nuclei were counterstained with hematoxylin for bright-field images or Hoechst for fluorescence (Thermo Fisher Scientific, 33342). Slides were mounted using a 50:50 PBS-glycerol solution. Images were acquired with a Zeiss AxioImager with Apotome2 or Zeiss Axioscan Z1 slide scanner. Images were minimally processed for global levels and white balance using Affinity Photo and Affinity Designer. Image settings and processing were identical across genotypes.

Quantifications were performed on scanned whole adrenals (Zeiss Axioscan scanner, 20× images) using the QuPath software version 0.3.1 (*72*). Briefly, annotations were made of whole adrenals or just the adrenal cortex, and the positive cell detection feature was used to identify positive cells. The threshold for identifying positive cells was set to avoid quantification of background on each image.

For quantification of phagocytosis, confocal images were acquired on a Zeiss LSM 800 Airyscan confocal microscope with ×40 magnification. Phagocytic events were identified and counted as the presence of steroidogenic cell markers (3βHSD or SF-1) within the boundaries of macrophages, defined by IBA-1 or MERTK staining. This was evaluated by a single operator, by manually scanning through z-stacks of 10 ×40 images per adrenal. The operator was blinded to the genotype.

### SA-βGal staining

SA-βGal staining was conducted following the protocol of (*4*, *73*) on frozen adrenal 10-μm sections. After drying for 15 min under a vacuum, the sections were rehydrated with PBS and then incubated overnight at 37°C in a humid atmosphere in a pH 6.0 staining solution composed of 7.4 mM citric acid, 25.3 mM dibasic sodium phosphate, 5 mM K_4_[Fe(CN)_6_], 5 mM K_3_[Fe(CN)_6_], 150 mM sodium chloride, 2 mM MgCl_2_, and X-Gal (1 mg/ml). Slides were mounted using a 50:50 PBS-glycerol solution and imaged on a Zeiss ApoTome microscope with an AxioCam MRm camera and/or a Zeiss Axioscan scanner.

### RNAScope RNA in situ hybridization

RNA in situ hybridizations were conducted on 5-μm paraffin sections using an RNAScope probe detecting *Mus musculus Cx3cl1* (#426211) with the RNAScope 2.5 HD Detection Kit (Brown), according to the manufacturer’s instructions (ACD Bio-Techne).

### Testosterone supplementation experiment

Testosterone or placebo implants were placed under gas anesthesia in the interscapular region of 4-week-old *Znrf3 cKO* female mice for 60 days. These testosterone implants (T-M/60, Belma) are designed to release daily doses of testosterone (from 51.9 to 154.5 μg/24 hours for plasma concentrations of 0.9 to 3.7 ng/ml) to produce physiological plasma concentrations in mice.

### Pexidartinib experiment

Chow was purchased from SAFE Nutrition Services (Augy, France). Male control and ZKO mice were fed either control chow (E8220A01R 00000 v0025 A04 Pur) or pexidartinib chow [E8220A01R 00000 v0398 A04 + pexidartinib (0.29 g/kg)] from 3 to 12 weeks of age. Pexidartinib (HY16749) was purchased from MedChemExpress and incorporated in the chow by SAFE Nutrition Services. Chow was replaced every 3 to 4 days, renewed weekly, and stored at 4°C when not in use.

### Fluorescence-activated cell sorting

Adrenals were harvested, and excess fat was removed under a dissecting microscope. Adrenals were immediately placed into 900 μl of digestion medium (table S2) and placed on ice until the end of the harvest. Adrenals were digested by incubating with a thermomixer set at 37°C, 900 rpm, for 37 min, stopping to pipette up and down at 10, 20, 30, 35, and 37 min. Digested samples were filtered through a 100-μm nylon mesh and centrifuged at 400*g* for 5 min at 4°C. Cells were resuspended in wash buffer [2.5 mM PBS-EDTA, deoxyribonuclease (100 μg/ml), and 0.5% bovine serum albumin] and stained appropriately. Cells were stained with Fixable Near-IR LIVE/DEAD stain (L34975, Invitrogen) for 30 min at room temperature, blocked with CD16/CD32 and TrueStain (426102, BioLegend) for 15 min at room temperature, and stained with the appropriate antibody panel for 20 min at room temperature (table S3). All staining/blocking steps were preceded and followed by wash steps, which included centrifugation at 200*g* for 4 min, followed by resuspension of the pellet with either wash buffer or the appropriate solution. Cells were immediately analyzed on the Attune NxT Flow Cytometer (reference no. A24858). Detailed analyses of the results were done using FlowJo software.

### Reverse transcription quantitative PCR

Adrenals were flash-frozen and stored at −80°C after harvest. RNAs were extracted using the Macherey-Nagel Nucleospin RNA Kit (reference no. 740955.250). After reverse transcription of 500 ng of total RNAs, complementary DNAs (cDNAs) were diluted 1:10 and PCRs were conducted using SYBR qPCR Premix Ex Taq II Tli RNase H+ (TAKRR820W, Takara). Primers can be found in table S4. Relative expression was calculated using the 2^−ΔΔ*C*T^ method.

### RNA sequencing for gene expression analysis

### Library preparation and sequencing

RNA sequencing was performed by the GenomEast platform, a member of the “France Genomique” consortium (ANR-10-INBS-0009). Library preparation was performed using TruSeq Stranded mRNA (reference guide, PN 1000000040498). RNA sequencing libraries were generated from 300 ng of total RNA using the TruSeq Stranded mRNA Library Prep Kit and IDT for Illumina TruSeq RNA UD Indexes (96 indexes, 96 samples) (Illumina, San Diego, USA), according to the manufacturer’s instructions. Briefly, following purification with poly-T oligo-attached magnetic beads, the mRNA was fragmented using divalent cations at 94°C for 2 min. The cleaved RNA fragments were copied into first-strand cDNA using reverse transcriptase and random primers. Strand specificity was achieved by replacing 3′-deoxythymidine 5′-triphosphate (dTTP) with deoxyuridine triphosphate nick end labeling (dUTP) during second-strand cDNA synthesis using DNA polymerase I and ribonuclease (RNase) H. Following addition of a single “A” base and subsequent ligation of the adapter on double-stranded cDNA fragments, the products were purified and enriched with PCR (30 s at 98°C; [10 s at 98°C, 30 s at 60°C, 30 s at 72°C] × 12 cycles; 5 min at 72°C) to create the cDNA library. Surplus PCR primers were further removed by purification using SPRI select beads (Beckman Coulter, Villepinte, France), and the final cDNA libraries were checked for quality and quantified using capillary electrophoresis. Libraries were sequenced on an Illumina HiSeq 4000 sequencer as single-read 50 base reads. Image analysis and base calling were performed using RTA version 2.7.7 and bcl2fastq version 2.20.0.422.

#### Genome mapping and differential gene expression analyses

Reads were filtered and trimmed to remove adapter-derived or low-quality bases using cutadapt v3.2 and checked again with FASTQC v0.11.7. Illumina reads were aligned to mouse reference genome (mm10) with Hisat2 v2.2.1. Read counts were generated for each annotated gene using R function “SummarizeOverlaps(),” and reads per kilobase of transcript per million reads mapped (RPKM) were calculated for each gene. Differential expression analysis with multiple testing correction was conducted using the R Bioconductor DESeq2 package v1.34.0. Raw (fastq) and processed data were deposited on Gene Expression Omnibus under accession no. GSE202940.

#### Generation of heatmaps

Heatmaps to represent differential gene expression were generated with the Biobase and gplots packages in R. They represent median-centered RPKM levels. Genes are sorted either by log_2_ fold change or by unsupervised clustering.

### Reanalysis of single-cell sequencing of adult mouse adrenals

The Seurat R package (*72*) was used to perform clustering analysis of single-cell data from Lopez *et al.* (*51*), available in the Gene Expression Omnibus GSE161751 (control adrenals from 10-week-old male mice). Raw sequencing data and annotated gene–barcode matrices were used for the input. Cells with more than 20 genes and genes expressed in more than three cells were selected for further analysis. After studying the distribution of count depth, number of genes, and mitochondrial read fraction, low-quality cells with less than 1000 counts, less than 400 genes detected, and percentage of mitochondrial gene counts higher than 25% were removed. Gene expression in each cell was then normalized by the total number of counts in the cell, multiplied by 10,000 to get counts per 10,000 (TP10K), and log-transformed to report gene expression as *E* = log(TP10K + 1).

The top 2000 highly variable genes with a *z*-score cutoff of 0.5 were then centered and scaled to have a mean of zero and SD of 1 and used as inputs for initial principal components analysis. The number of principal components was chosen according to the PCElbowPlot function and JackStrawPlot function. Next, the Louvain algorithm implemented in Seurat was used to iteratively group cells together, with the goal of optimizing the standard modularity function. The resolution parameter for clustering was set at *r* = 1. The default Wilcoxon rank sum test was used by running FindAllMarkers function in Seurat to find differentially expressed markers in each cluster. Last, each cell type was annotated after extensive literature reading and searching for specific gene expression patterns. Violin plot representations were used for visualizing expression of the different markers.

### TCGA-ACC data

TCGA gene expression and clinical ACC data were extracted from the TCGA database. Distribution in the good (C1B) and poor prognosis (C1A) groups was previously defined on the basis of unsupervised clustering (*27*). Expression data were standardized by the relative standard error of the mean (RSEM) algorithm and transformed into log_2_ to refocus and symmetrize values’ distribution. The macrophage signature was defined as the mean expression (*z* score) of *CD74*, *CXCL2*, *CCL4*, *APOE*, *CCL3*, *CTSS*, *C1QA*, *C1QB*, *C1QC*, and *AIF1*. These were found as highly up-regulated genes in macrophages in scRNA-seq analyses of adult mouse adrenals (see above) (*51*). For GSEA, TCGA-ACC patients were dichotomized on the basis of the expression of a phagocytic signature (*z* score of *TYROBP*, *TREM2*, and *CD68*) with patients classified as high (expression above median) or low (expression below median). Differential gene expression between patients from the phagocytic-high and phagocytic-low groups was computed using the limma R package. The volcano plot representing differential expression between these two groups was generated in R with the calibrate library. Kaplan-Meier analysis was conducted in GraphPad Prism after dichotomization of patients according to expression of the phagocytic signature.

### Gene set enrichment analyses

GSEAs were conducted on gene expression data from mouse models and TCGA-ACC patients, using GSEA 4.1.0 with gene sets from the MSigDB and MGI GO databases and with custom-curated gene sets (table S5). Permutations were set to 1000 and performed on gene sets. Phagocytosis gene sets were curated from an extensive search of the literature, including papers by Park and Kim (*73*), Lecoultre *et al.* (*74*), and Janda *et al.* (*75*), and extracted from the MGI GO database. Senescence gene sets were extracted from papers by Eggert *et al.* (*37*), Kuilman *et al.* (*76*), Özcan *et al.* (*77*), Acosta *et al.* (*78*), Fridman and Tainsky (*79*), Coppé *et al.* (*80*, *81*), Buhl *et al.* (*82*), and Saul *et al.* (*83*). LM22 and ImmuCC gene sets were derived from gene expression signatures published by Newman *et al.* (*84*) and Chen *et al.* (*32*). To reduce the gene expression matrix into simple gene identifier lists for GSEA, genes in each of the lists were attributed to their cognate immune cell type based on their maximum of expression across all cell types. This resulted in gene signatures for each immune cell type that were then used in GSEA (table S5). M0, M1, and M2 macrophage gene sets were further concatenated to result in global LM22 and ImmuCC macrophage gene sets. The mouse adrenal macrophage gene set was defined as the 100 most significantly up-regulated genes within the two macrophage clusters (compared to all other clusters) in our reanalysis of the single-cell sequencing study of adult mouse adrenals by Lopez *et al.* (*51*). The cytokine gene set was curated from an extensive search of the literature. NFκB and DNA replication gene sets were extracted from MSigDB C2, Hallmarks, and C5 datasets.

GSEA output was either displayed as dot plots or enrichment curves. Dot plots represent the normalized enrichment score and FDR [size of dots defined as −log_10_(FDR)] and were drawn using the ggplot2 library in R. Enrichment curves were drawn by feeding GSEA output to the GSEA_replot R function, developed by T. Kuilman (https://github.com/PeeperLab/Rtoolbox/blob/master/R/ReplotGSEA.R). Dot plots and enrichment curves were further processed in Affinity Designer for color matching and superimposition.

### CIBERSORTx and mMCP analyses

CIBERSORTx (*31*) analyses were run on the CIBERSORTx server (https://cibersortx.stanford.edu) using the LM22 matrix and a mixture file representing gene expression data in control and *Znrf3 cKO* adrenals at 4, 6, and 12 weeks or TCGA-ACC patients’ data, dichotomized on the basis of high or low expression of the phagocytic signature (see TCGA-ACC data). Output of CIBERSORTx was then processed in R to concatenate subpopulations of macrophages, B cells, CD4 T cells, NK cells, dendritic cells, and mast cells. Ggplot2 was then used to generate stacked bar plots representing the percentage of each immune cell population. Statistical analyses between genotypes or patients’ groups were computed using the Mann-Whitney test.

mMCP analyses were run in R using the mMCP counter package (https://github.com/cit-bioinfo/mMCP-counter), following instructions by Petitprez *et al.* (*33*). Stacked bar plots were generated by ggplot2, and statistical analyses were conducted as above.

### Statistical analyses

Minimal sample size was set at *n* = 3 allowing for detection of 40% increases/decreases with α = 0.05, δ = 0.4, and SD = 1.0. Statistical analyses were conducted with R and GraphPad Prism 9. Normality of data was assessed using D’Agostino and Pearson normality test. Statistical analyses were performed by two-tailed Student’s *t* test (two groups with normal distribution) or two-tailed Mann-Whitney test (two groups without normal distribution). Multiple comparisons were analyzed by two-way analysis of variance (ANOVA), followed by Sidak’s multiple comparisons test. All bars represent means ± SEM.

## Notes

View/request a protocol for this paper from *Bio-protocol*.
