# Supplementary material for: Sexually dimorphic activation of innate antitumor immunity prevents adrenocortical carcinoma development
Source: Sci Adv. 2022 Oct 14;8(41):eadd0422. doi: 10.1126/sciadv.add0422 (PMC9565812; doi:10.1126/sciadv.add0422)
Supplement: Supplementary file 1 — Figs. S1 to S8 [file sciadv.add0422_sm.pdf]

Science Advances
AAAS

Supplementary Materials for

Sexually dimorphic activation of innate antitumor immunity prevents adrenocortical carcinoma development

James J. Wilmouth Jr. *et al.*

Corresponding author: Pierre Val, pierre.val@uca.fr

Sci. Adv. **8**, eadd0422 (2022)
DOI: 10.1126/sciadv.add0422

The PDF file includes:

Figs. S1 to S8
Legends for tables S1 to S5

Other Supplementary Material for this manuscript includes the following:

Tables S1 to S5

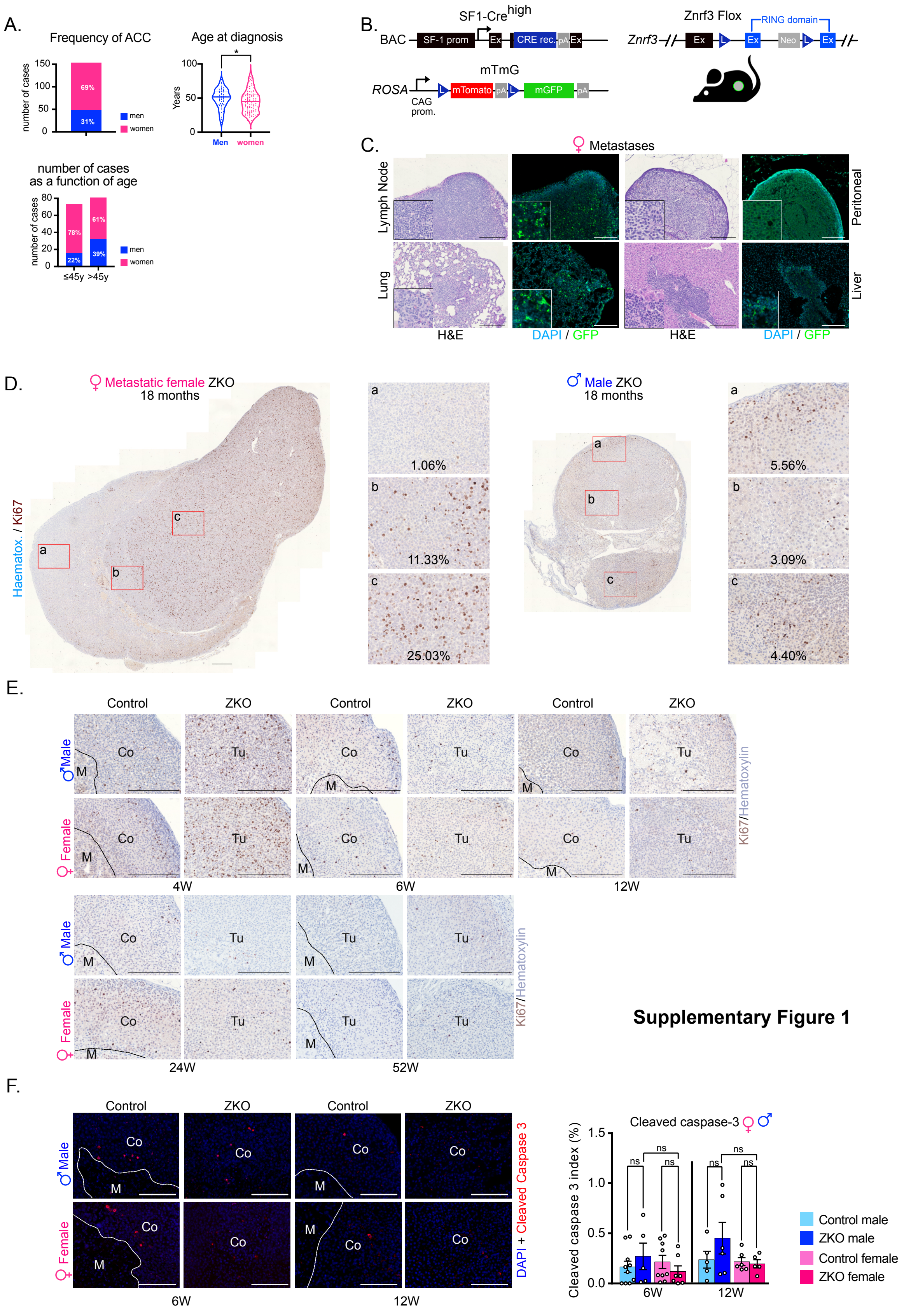

Supplementary Figure 1

Fig. S1. Sexual dimorphism in ACC patients and kinetic characterisation of *Znrf3 cKO* mice. **A-** Demographics of the TCGA cohort of ACC showing overall frequency of ACC in men and women, age at diagnosis as a function of sex and number of cases as a function of age and sex. **B-** *Znrf3 cKO* mice were obtained by mating mice with LoxP sites flanking the two exons that encode the RING domain of the protein (28) with Sf1-Cre^{high} mice, in which expression of the Cre recombinase is under the control of the regulatory regions of the *Sf1* gene (68). For experiments that did not involve flow cytometry, the mTmG reporter was also included. It allows lineage tracing of recombined cells by expression of a membrane GFP, following Cre-mediated excision of the membrane Tomato cassette, which is normally expressed in non-recombined cells (69). **C-** Histological (H&E) and immunohistochemical (GFP) analysis of metastases from primary adrenal tumours to lymph nodes, lungs, peritoneal cavity and liver. GFP staining allows identification of tumour cells through SF-1:Cre-mediated recombination of the mTmG locus within the primary tumour. **D-** Immunohistochemical analysis of Ki67 expression in the primary adrenal tumour of 18-month-old *Znrf3 cKO* metastatic female (left) and non-metastatic male (right). The two tumours are represented at the same scale. Insets show heterogeneous proliferation in female tumour and more homogeneous proliferation in male tumour. **E-** Kinetic immunohistochemical analysis of Ki67 expression in control males/females and *Znrf3 cKO* males/females from 4 to 52 weeks. **F-** Immunohistochemical analysis (left panels) and quantification of cleaved-caspase 3 expression (graph) in control males/females and *Znrf3 cKO* males/females at 4 and 12 weeks. Co: cortex; Tu: tumour. Scale bar = 200 µm (B-F). Graphs represent mean +/- SEM. Statistical analyses were conducted by Mann-Whitney tests in A and by 2-way ANOVA in E ns: not significant; * $p<0.05$.

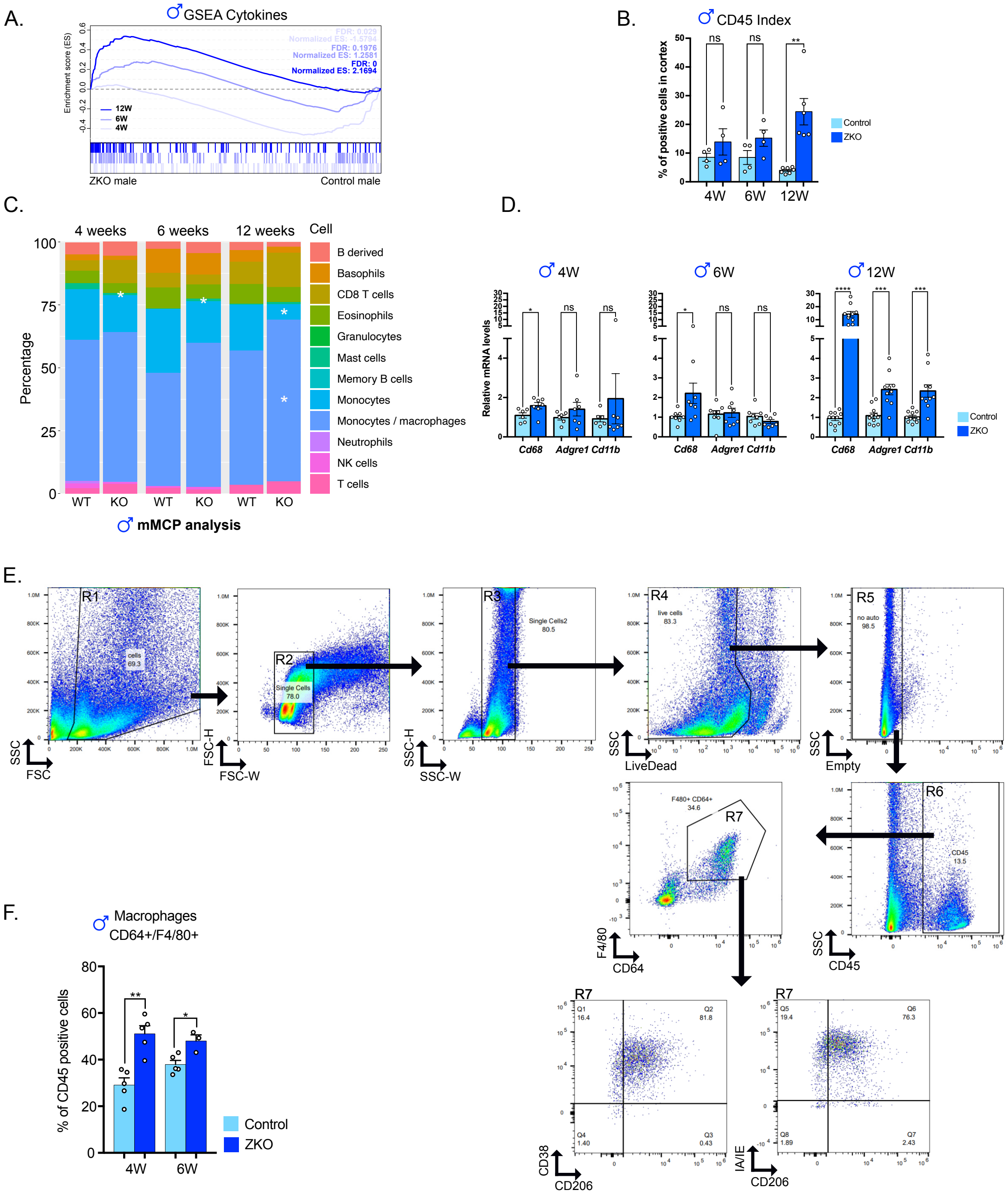

Supplementary Figure 2

Fig. S2. Characterization of immune infiltration in *Znrf3 cKO* male mice. A- GSEA of gene expression from 4, 6 and 12 control and *Znrf3 cKO* males. The plot represents enrichment of cytokines gene set in *Znrf3 cKO* compared with controls adrenals. **B-** Quantification of the CD45+ index as the ratio of CD45-positive cells over total nuclei in the cortex of male control and *Znrf3 cKO* mice at 4, 6 and 12 weeks. **C-** Stacked bar plots representing immune cell populations deconvoluted using the mMCP algorithm from gene expression data in control and *Znrf3 cKO* adrenals at 4, 6 and 12 weeks. **D-** RTqPCR analysis of the expression of macrophages-related genes in control and *Znrf3 cKO* males at 4, 6 and 12 weeks. **E-** Flow cytometry gating strategy to identify macrophages in adrenals from control and *Znrf3 cKO* mice. The first gate (R1) was drawn to identify cells of interest and exclude debris. Cells were then gated to exclude doublets (R2 & R3) and live cells were identified using a Live/Dead stain (R4). Live cells were plotted against an empty channel to gate-off auto-fluorescent cells (R5). F4/80+ CD64+ macrophages (R7) were then identified from CD45+ cells (R6). From the macrophage population, M1 macrophage markers CD38 & MHC II (IA/IE) were used against the M2 marker CD206 to identify potential M1 vs M2 polarization. The provided example is from a control male at 4 weeks of age. **F-** Quantification of macrophages $CD64^{+}/F4/80^{+}$ as a percentage of $CD45^{+}$ live cells in control and *Znrf3 cKO* male adrenals at 4 and 6 weeks. Right panel, quantification of absolute numbers of macrophages by flow cytometry. Graphs represent mean +/- SEM. Statistical analyses in B, C, D and F were conducted by Mann-Whitney tests. ns: not significant; * $p<0.05$; ** $p<0.01$; *** $p<0.001$; **** $p<0.0001$.

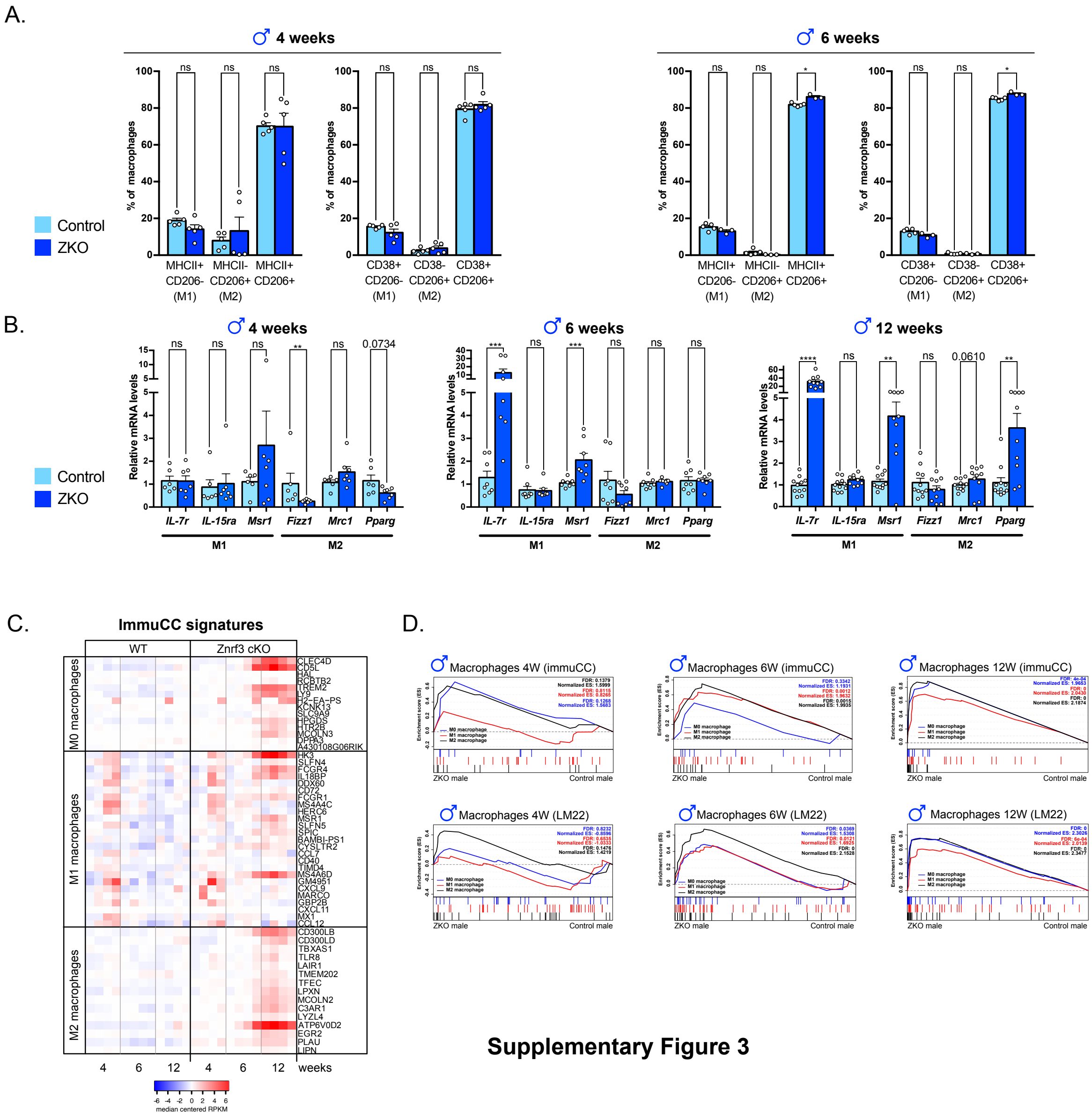

Supplementary Figure 3

Fig. S3. Characterization of macrophages in *Znrf3 cKO* male mice. A- Quantification of macrophages sub-populations by flow cytometry analysis of control and *Znrf3 cKO* male adrenals at 4 (left panels) and 6 weeks (right panels). **B-** RTqPCR analysis of the expression of M1 and M2 macrophages-related genes in control and *Znrf3 cKO* males at 4, 6 and 12 weeks. **C-** Heatmap showing expression of M0, M1 and M2 gene signatures (extracted from the ImmuCC dataset) in RNA sequencing data from control and *Znrf3 cKO* males at 4, 6 and 12 weeks. All genes in the datasets are represented **D-** GSEA of M0, M1 and M2 macrophages gene sets from the ImmuCC and LM22 datasets in male *Znrf3 cKO* adrenals compared with control adrenals at 4, 6 and 12 weeks. Graphs represent mean +/- SEM. Statistical analyses in A and B were conducted by Mann-Whitney tests. ns: not significant; * $p<0.05$; ** $p<0.01$; *** $p<0.001$; **** $p<0.0001$.

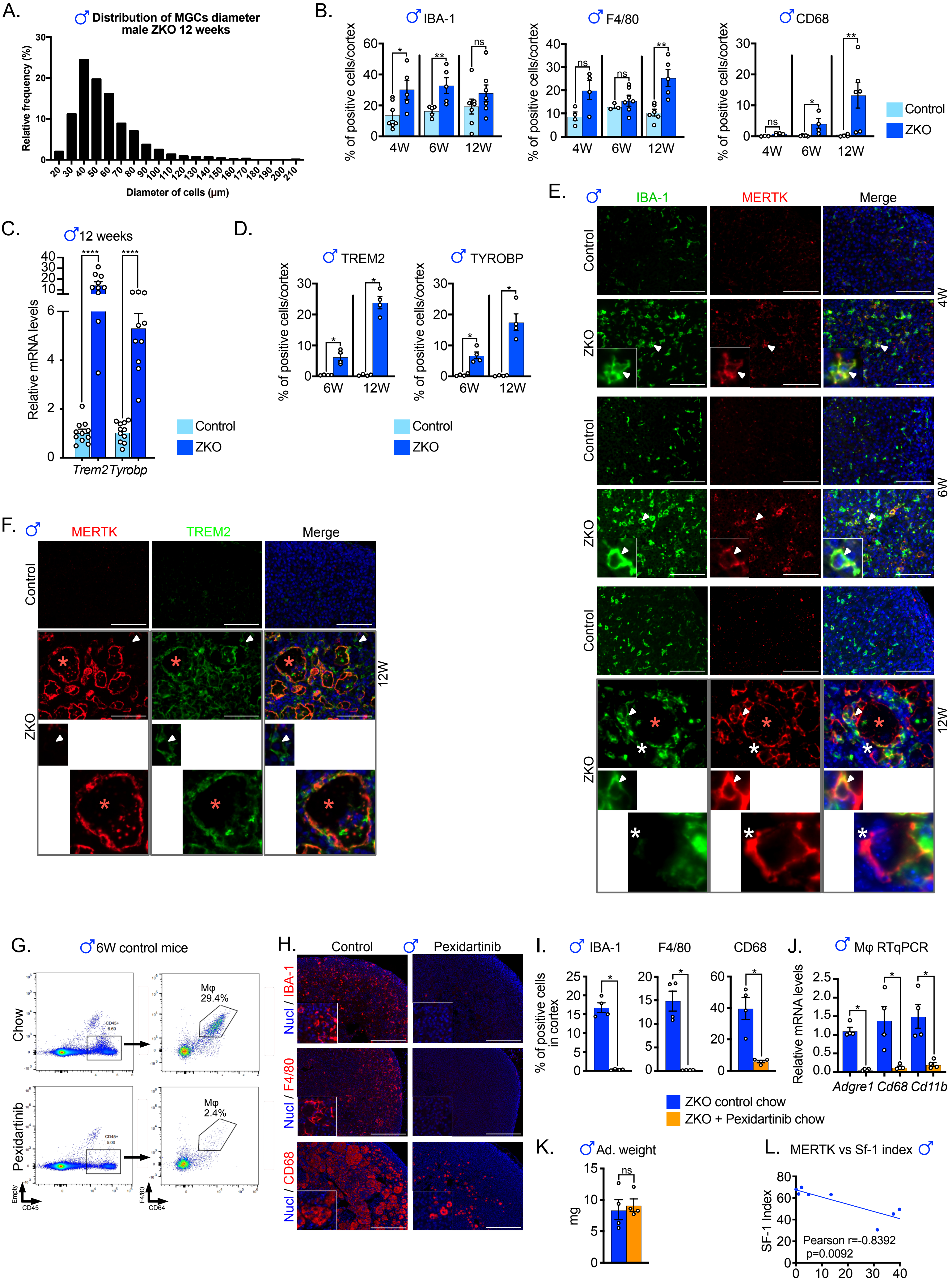

Supplementary Figure 4

Fig. S4. Characterization of multinucleated giant cells and impact of Pexidartinib treatment on macrophages. A- Diameter distribution of multinucleated giant cells in 12-week-old male *Znrf3 cKO* adrenals (n=5). **B-** Quantification of the IBA-1, F4/80 and CD68+ index as the ratio of positive cells over total nuclei in the cortex of male control and *Znrf3 cKO* mice at 4, 6 and 12 weeks. **C-** RTqPCR analysis of *Trem2* and *Tyrobp* expression in 12-week-old control and *Znrf3 cKO* male adrenals. **D-** Quantification of the TREM2 and TYROBP+ index as the ratio of positive cells over total nuclei in the cortex of male control and *Znrf3 cKO* mice at 6 and 12 weeks. **E-** Immunohistochemical analysis of IBA-1 and MERTK expression in control and *Znrf3 cKO* adrenals at 4, 6 and 12 weeks. Arrowheads show double-positive macrophages. White stars show MERTK-positive, IBA-1-negative mononucleated macrophages. Red stars show MERTK-positive, IBA-1-negative multinucleated macrophages. **F-** Immunohistochemical analysis of MERTK and TREM2 expression in control and *Znrf3 cKO* adrenals at 12 weeks. Arrowheads show double-positive mononucleated macrophages. Red stars show MERTK-positive, TREM2-positive multinucleated macrophages. **G-** Flow cytometry analysis of CD45+/CD64+/F4/80+ macrophages in the adrenals of 6-week-old control male mice fed for one week with a standard chow (top panels) or a chow enriched with 290 mg/kg of Pexidartinib. **H-** Immunohistochemical analysis of IBA-1, F4/80 and CD68 expression in male *Znrf3 cKO* mice treated with either control chow or pexidartinib-enriched chow (290 mg/kg) from 3 to 12 weeks. **I-** Quantification of the IBA-1, F4/80 and CD68+ index as the ratio of positive cells over total nuclei in the cortex of male *Znrf3 cKO* mice treated with control or pexidartinib-enriched chow. **J-** RTqPCR analysis of *Adgre1, Cd68* and *Cd11b* expression in the adrenals of *Znrf3 cKO* mice treated with control or pexidartinib-enriched chow. **K-** Adrenal weight in male *Znrf3 cKO* mice treated with either control chow or pexidartinib-enriched chow (290 mg/kg) from 3 to 12 weeks. **L-** Analysis of the correlation between MERTK and SF1-positive cell- indexes in *Znrf3 cKO* mice treated with either control chow or pexidartinib-enriched chow (290 mg/kg) from 3 to 12 weeks. Scale bar = 100 µm (C-D); 200µm (F). Graphs in B, C, D, I, J and K represent mean +/- SEM. Statistical analyses were conducted by Mann-Whitney tests. ns: not significant; * $p<0.05$; ** $p<0.01$; *** $p<0.001$; **** $p<0.0001$.

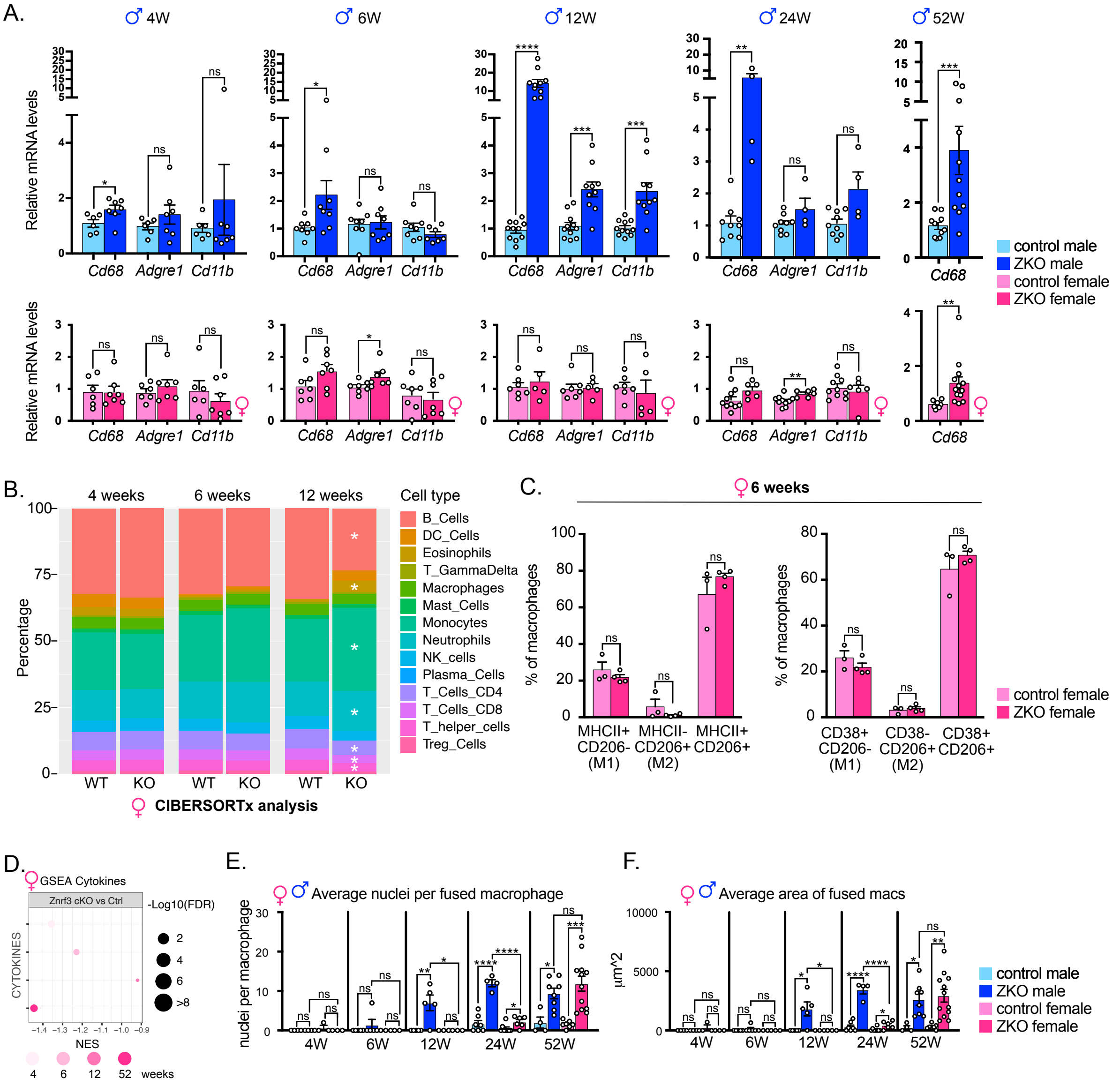

Supplementary Figure 5

Fig. S5. Characterization of immune infiltration in *Znrf3 cKO* female mice, compared with males. A- RTqPCR analysis of the expression of macrophages-associated genes in control and *Znrf3 cKO* males (top panels) and control and *Znrf3 cKO* females (bottom panels) from 4 to 52 weeks. **B-** Stacked bar plots representing immune cell populations deconvoluted using CIBERSORTx and the LM22 expression matrix, from gene expression data in control and *Znrf3 cKO* female adrenals at 4, 6 and 12 weeks. **C-** Quantification of macrophages sub-populations by flow cytometry analysis of control and *Znrf3 cKO* female adrenals at 6 weeks. **D-** GSEA of gene expression from 4, 6, 12 and 52-week-old control and *Znrf3 cKO* females. The plot represents enrichment of cytokines gene set in *Znrf3 cKO* compared with control adrenals. **E-** Analysis of the number of nuclei per fused macrophages, determined on H&E sections of control and *Znrf3 cKO* male and female adrenals from 4 to 52 weeks. **F-** Average area of fused macrophages (µm^2) determined on H&E sections of control and *Znrf3 cKO* male and female adrenals from 4 to 52 weeks. Graphs in A, C, E and F represent mean +/- SEM. Statistical analyses were conducted by Mann-Whitney tests in A, B and C and by two-way ANOVA in E and F. ns: not significant; * $p<0.05$; ** $p<0.01$; *** $p<0.001$; **** $p<0.0001$.

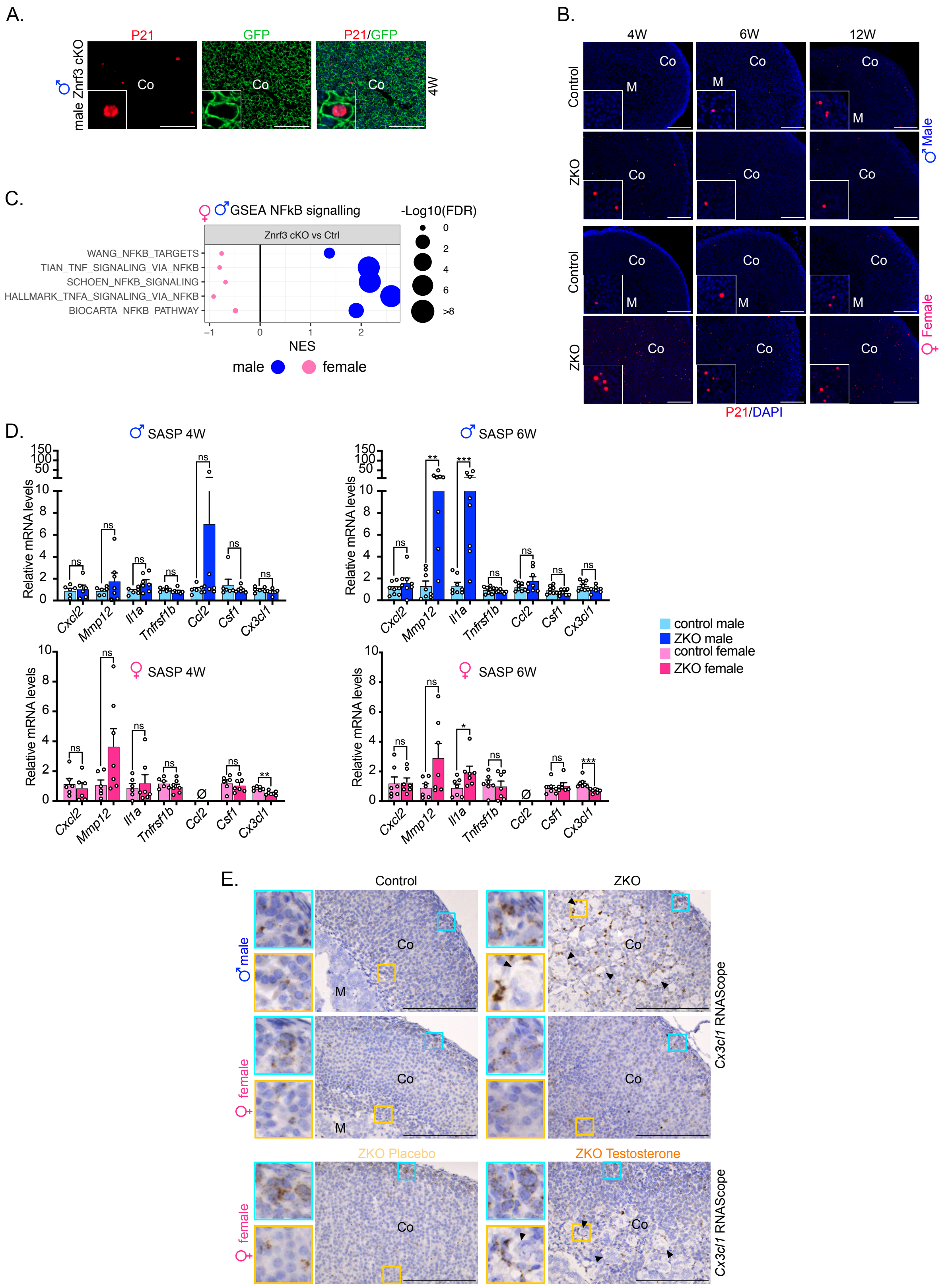

Supplementary Figure 6

Fig. S6. Characterization of senescence and SASP in *Znrf3 cKO* mice. A- Immunohistochemical analysis of P21 and GFP (marking SF-1:Cre-mediated recombination of mTmG in steroidogenic cells) in 6-week-old male *Znrf3 cKO* adrenals. **B-** Immunohistochemical analysis of P21 expression in control and *Znrf3 cKO* male and female adrenals at 4, 6 and 12 weeks. **C-** GSEA of gene expression from 12-week-old control and *Znrf3 cKO* males and females. The plot represents enrichment of NFkB-related gene sets in *Znrf3 cKO* compared with control adrenals (sex matched). **D-** RTqPCR analysis of the expression of SASP-associated genes in control and *Znrf3 cKO* males (top panels) and control and *Znrf3 cKO* females (bottom panels) at 4 and 6 weeks. **E-** Expression of *Cx3cl1* mRNA analysed by RNAScope^{TM} on sections of 12-week-old control and *Znrf3 cKO* males and females (top panels) and 12-week-old placebo and testosterone-treated *Znrf3 cKO* females (bottom panels). One external area of the cortex (blue box) and one area of the internal cortex, where fused macrophages reside, (yellow box) are shown enlarged on the left size of the corresponding images. Black arrowheads show fused macrophages. Co: cortex; M: medulla. Scale bar = 100 µm (A); 200 µm (B, E). Co: cortex; M: medulla. Graphs in D represent mean +/- SEM. Statistical analyses were conducted by Mann-Whitney tests. ns: not significant; * $p<0.05$; ** $p<0.01$; *** $p<0.001$.

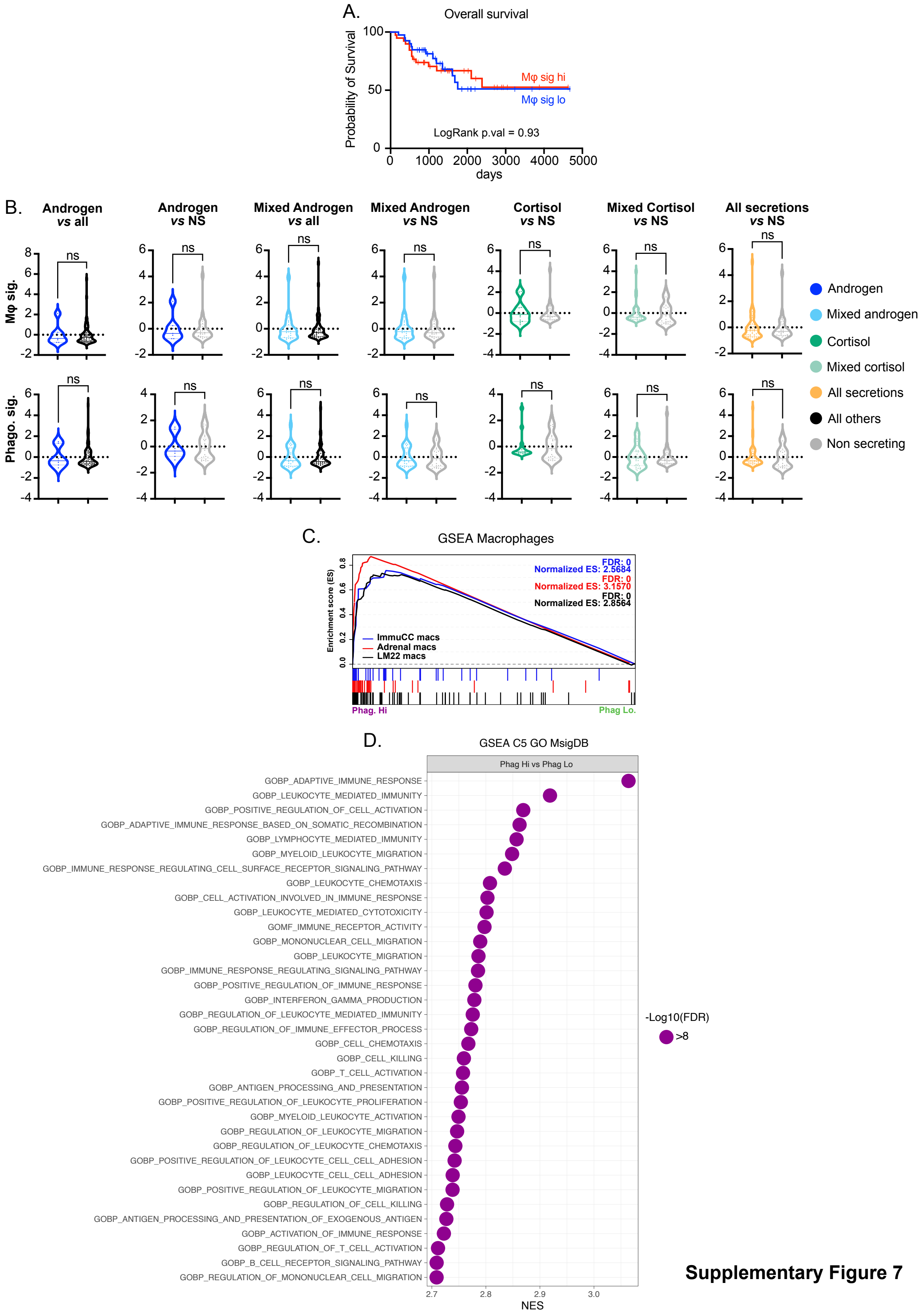

Supplementary Figure 7

Fig. S7. Analysis of macrophage signatures in TCGA-ACC patients. A- Survival analysis of patients of the TCGA program dichotomised as patients with high (red) or low (blue) expression of the macrophage signature. **B-** Expression of a global (top panels) and phagocytic (bottom panels) macrophage gene signature in ACC patients from the TCGA program with respect to hormone secretion (NS: non-secreting tumour). **C-** GSEA of macrophages gene sets in TCGA ACC patients with high expression of the phagocytic signature, compared with patients with low phagocytic signature. **D-** GSEA of gene expression from TCGA ACC patients. The plot represents the top 35 enriched gene sets from the C5 Gene Ontology database (MSigDB), in patients with high expression of the phagocytic signature, compared with patients with low phagocytic signature. Statistical analyses in B were conducted by Mann-Whitney tests. ns: not significant.

A.

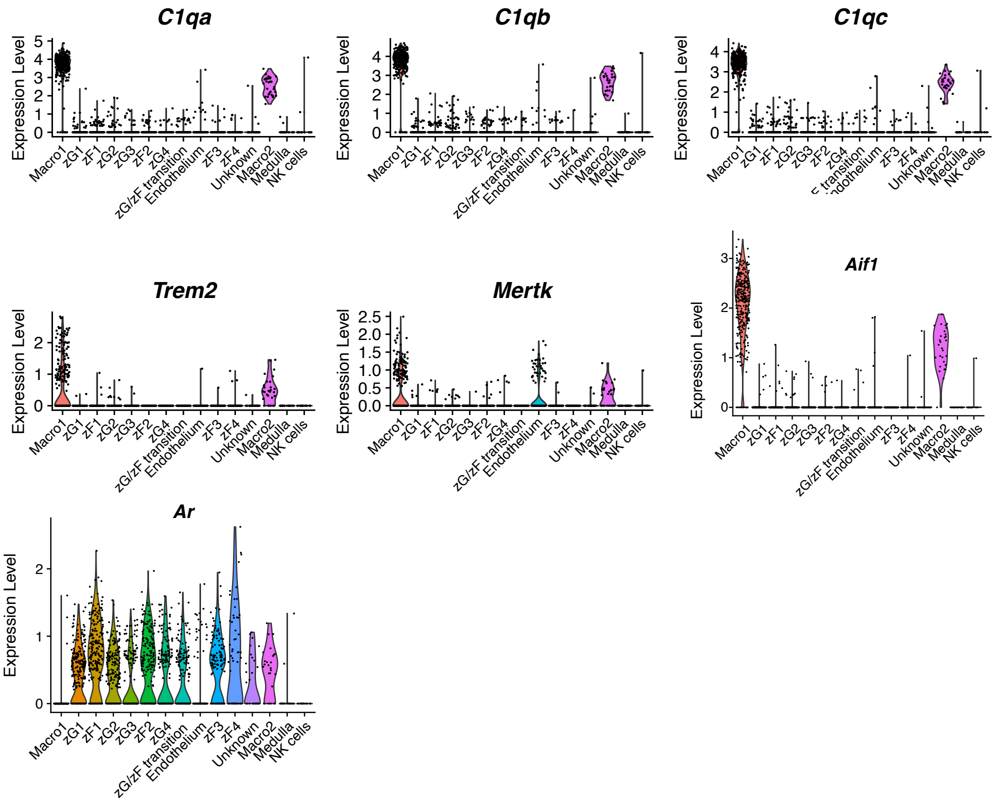

B.

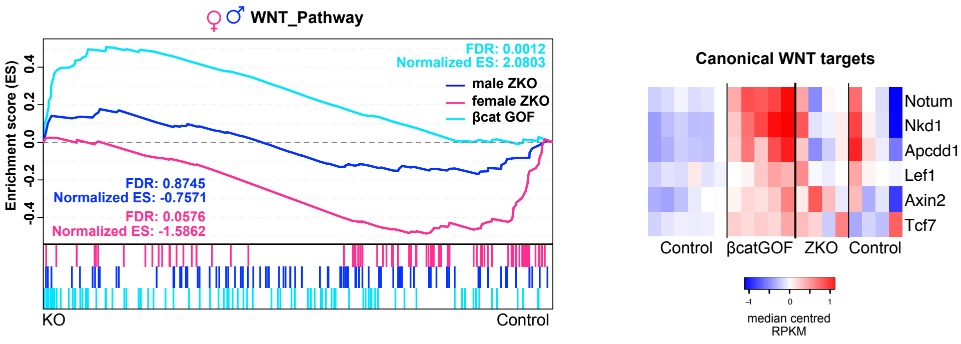

Supplementary Figure 8

Fig. S8. Gene expression in adrenal macrophages and canonical WNT pathway activation in mouse models. A- Expression of macrophages markers and Androgen Receptor (*Ar*) in single-cell RNA sequencing data from 10-week-old adult male mouse adrenals **B-** Left panel, GSEA of canonical WNT pathway genes in β-catenin gain of function mice (βcatGOF) (*64*) and 12-week-old male and female *Znrf3 cKO* mice compared with controls. Right panel, heatmap showing expression of canonical WNT pathway target genes in control, βcatGOF and 12-week-old *Znrf3 cKO* male mice.

Table S1. Conditions for immunohistochemistry.

Table S2. Protocol for adrenal tissue digestion before flow cytometry.

Table S3. Antibodies used in flow cytometry.

Table S4. Primers used for qPCR.

Table S5. Gene signatures used in GSEA analyses.
